# MEKK3 coordinates with FBW7 to regulate WDR62 stability and neurogenesis

**DOI:** 10.1371/journal.pbio.2006613

**Published:** 2018-12-19

**Authors:** Dan Xu, Minghui Yao, Yaqing Wang, Ling Yuan, Joerg D. Hoeck, Jingwen Yu, Liang Liu, Yvonne Y. C. Yeap, Weiya Zhang, Feng Zhang, Yinghang Feng, Tiantian Ma, Yujie Wang, Dominic C. H. Ng, Xiaoyin Niu, Bing Su, Axel Behrens, Zhiheng Xu

**Affiliations:** 1 State Key Laboratory of Molecular Developmental Biology, CAS Center for Excellence in Brain Science and Intelligence Technology, Institute of Genetics and Developmental Biology, Chinese Academy of Sciences, Beijing, China; 2 College of Biological Science and Engineering, Institute of Life Sciences, Fuzhou University, Fuzhou, China; 3 Center for Medical Genetics, School of Life Sciences, Central South University, Changsha, Hunan, China; 4 CR-UK London Research Institute, London, United Kingdom; 5 School of Biomedical Science, Faculty of Medicine, University of Queensland, St Lucia, Australia; 6 Sino-Danish College, University of Chinese Academy of Science, Beijing, China; 7 Shanghai Institute of Immunology, Shanghai Jiao Tong University School of Medicine, Shanghai, China; 8 Department of Immunobiology, Yale University School of Medicine, New Haven, Connecticut, United States of America; 9 Adult Stem Cell Laboratory, The Francis Crick Institute, London, United Kingdom; 10 King’s College London, Faculty of Life Sciences and Medicine, Guy’s Campus, London, United Kingdom; 11 Parkinson’s Disease Center, Beijing Institute for Brain Disorders, Beijing, China; University of Southern California Keck School of Medicine, United States of America

## Abstract

Mutations of *WD repeat domain 62* (*WDR62*) lead to autosomal recessive primary microcephaly (MCPH), and down-regulation of WDR62 expression causes the loss of neural progenitor cells (NPCs). However, how WDR62 is regulated and hence controls neurogenesis and brain size remains elusive. Here, we demonstrate that mitogen-activated protein kinase kinase kinase 3 (MEKK3) forms a complex with WDR62 to promote c-Jun N-terminal kinase (JNK) signaling synergistically in the control of neurogenesis. The deletion of *Mekk3*, *Wdr62*, or *Jnk1* resulted in phenocopied defects, including premature NPC differentiation. We further showed that WDR62 protein is positively regulated by MEKK3 and JNK1 in the developing brain and that the defects of *wdr62* deficiency can be rescued by the transgenic expression of *JNK1*. Meanwhile, WDR62 is also negatively regulated by T1053 phosphorylation, leading to the recruitment of F-box and WD repeat domain-containing protein 7 (FBW7) and proteasomal degradation. Our findings demonstrate that the coordinated reciprocal and bidirectional regulation among MEKK3, FBW7, WDR62, and JNK1, is required for fine-tuned JNK signaling for the control of balanced NPC self-renewal and differentiation during cortical development.

## Author summary

Microcephaly is a neural developmental disorder characterized by significantly reduced brain size and variable intellectual disability. *WD repeat domain 62 (WDR62)* was identified as the second most common gene for autosomal recessive primary microcephaly (MCPH) in human. Here, we studied the underlying regulatory mechanism of WDR62 and the impact on generation of new neurons. We show that *mitogen-activated protein kinase kinase kinase 3 (Mekk3)*, *Wdr62*, and *c-Jun N-terminal kinase 1 (Jnk1)* knockout (KO) mice have defects in the generation and maturation of neurons. We demonstrate that WDR62 stability is positively regulated by a mitogen-activated protein kinase kinase kinase (MAPKKK), MEKK3, but negatively regulated by the E3 ligase, F-box and WD repeat domain-containing protein 7 (FBW7). These positive and negative factors calibrate the strength of the activity of the JNK signaling pathway, which controls self-renewal and differentiation of neural progenitor cells (NPCs) during brain development. This finding improves our understanding of the molecular pathogenesis of MCPH.

## Introduction

Establishment of the mammalian neocortex requires precise control of proliferation and self-renewal of neural progenitor cells (NPCs), as well as the differentiation of NPCs and neuronal migration [1–4]. Defects in these processes lead to brain disorders, including autosomal recessive primary microcephaly (MCPH) [5–8]. During cortical development, the balance between symmetric and asymmetric cell division of NPCs determines the size of the NPC pool for ongoing neurogenesis and ultimately brain size [9–16]. Disturbance in symmetric cell division leads to a reduction in the NPC pool and a falloff of neuron production [10,14,16–19].

MCPH is a neural developmental disorder characterized by significantly reduced brain size and variable intellectual disability [20,21]. Most of the 23 MCPH-associated genes (*MCPH1-23*) identified so far are predicted to be associated with the mitotic apparatus, such as centrosomes or mitotic spindle poles, at least during part of the cell cycle [6,22–33].

*WD repeat domain 62* (*WDR62*) was identified as a causative gene of MCPH [22–24]. More than 50% of MCPH cases worldwide are caused by mutations in either *abnormal spindle-like microcephaly-associated* (*ASPM*) or *WDR62* [7,21]. WDR62 has been reported to be a scaffold protein for the c-Jun N-terminal kinase (JNK) signaling pathway by forming a complex with MAP kinase kinases (MKKs) 4 and 7, and JNKs [34,35], similar to what we and other groups have demonstrated for the JNK pathway scaffold proteins such as Plenty of SH3s (POSH) and JNK-interacting proteins (JIPs) [36–39]. We and others have recently shown that WDR62 plays a role in NPC maintenance [40–42]. However, how WDR62 and JNK signaling are regulated for the control of neurogenesis and brain size during brain development is still not clear.

Here, we have identified 2 novel WDR62-interacting proteins: the MAP3K kinase, mitogen-activated protein kinase kinase kinase 3 (MEKK3), and the E3 ubiquitin ligase, F-box and WD repeat domain-containing protein 7 (FBW7). Using in vivo short hairpin RNA (shRNA) knockdown (KD), gene knockout (KO), and transgenic mice, we find that MEKK3, WDR62, and JNK1 play an important role in neurogenesis during cortical development. We demonstrate further that there is synergy between MEKK3 and WDR62 in the activation of JNK signaling while FBW7 negatively regulates the stability of WDR62 through specific phosphorylation of WDR62. Taken together, our findings have revealed the detailed mechanism regulating WDR62 protein levels via interaction with MEKK3 and FBW7, to control proliferation and differentiation of NPCs during brain development. Our study thus unravels a novel molecular mechanism underlying MCPH pathogenesis.

## Results

### MEKK3 plays an important role in neurogenesis during neocortical development

**Fig 1:**
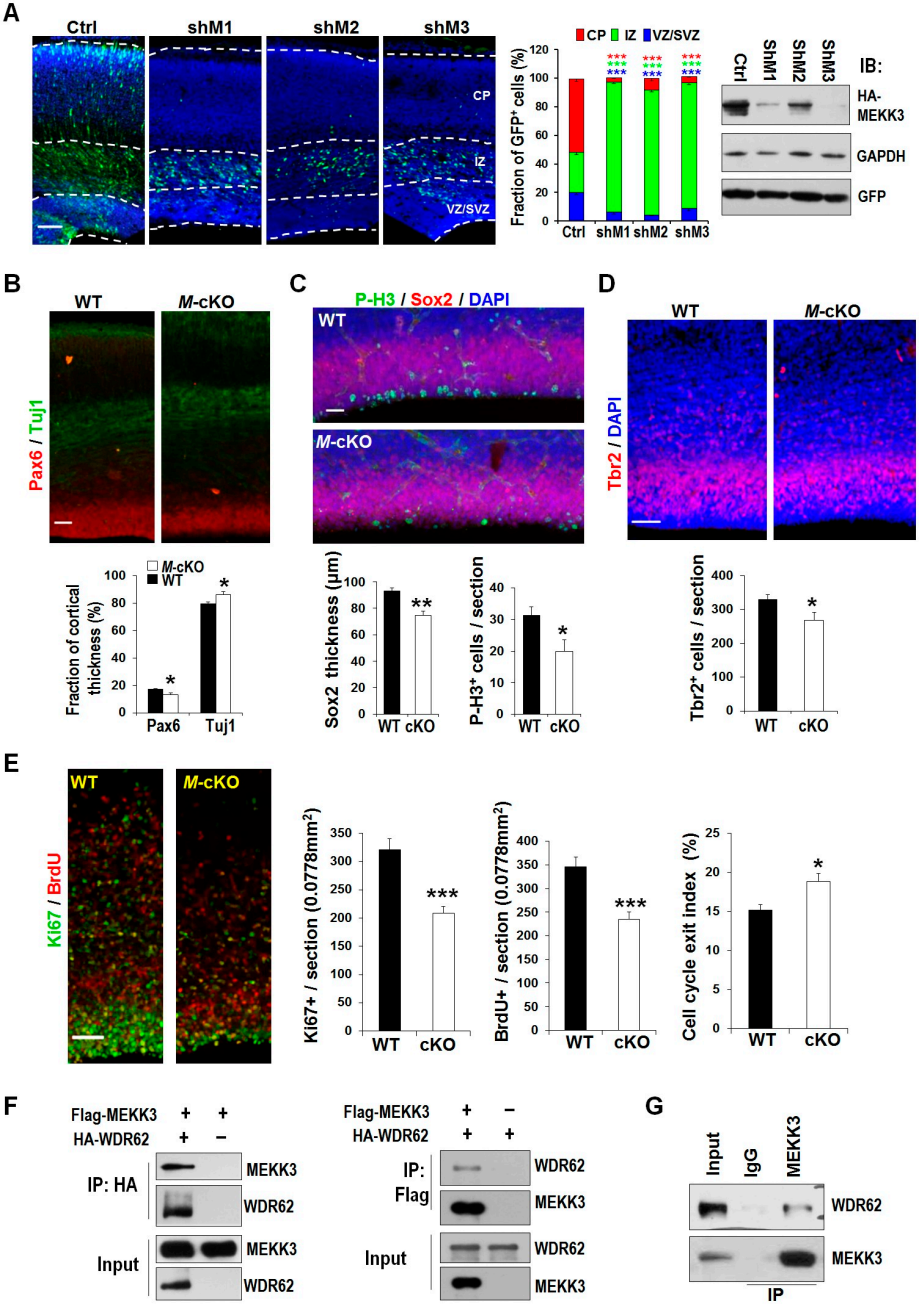
MEKK3 is critical for cortical neurogenesis. (A) Coronal sections of rat cortices electroporated in utero with bicistronic constructs encoding both EGFP and MEKK3 shRNAs (shM1, 2, or 3) or control shRNA (Ctrl) at E16.5 and inspected at E20.5. Middle panel: quantification of EGFP^+^ cell distribution. Data are means ± SEM; ***P < 0.0001; Ctrl, *n* = 9; shM1 and shM3, *n* = 6; shM2 *n* = 8. Right panel: KD efficiency of *Mekk3* shRNAs. HA-MEKK3 was cotransfected with shCtrl or shM into HEK293 cells; 24 hours later, cell lysates were analyzed by immunoblotting with anti-HA antibody, with GAPDH as a loading control and GFP to indicate transfection efficiency. (B–E) Coronal sections of E16.5 mice WT or *Mekk3* cKO (M-cKO) cortices stained with antibodies against different markers or DAPI as indicated. In panel E, cell-cycle exit index means percent of BrdU+/Ki67- “among all BrdU+”. All data are means ± SEM; *P < 0.05, **P < 0.01, ***P < 0.001. (B) WT and cKO, *n* = 4. (C) Left panel: WT, *n* = 5; cKO, *n* = 3; right panel: WT, *n* = 7; cKO, *n* = 3. (D) WT and cKO *n* = 5. (E) WT, *n* = 5; cKO, *n* = 6. *n*: number of brain slices from different brains. Scale bars: 100 μm (panel A), 50 μm (panels B–E). (F) MEKK3 interacts with WDR62. HEK293 cells were transfected with Flag-*MEKK3* and HA-*WDR62* cDNA, either alone or in combination. Cell lysates were immunoprecipitated with HA or Flag antibodies and probed with Flag and HA antibodies. (G) Endogenous WDR62 interacts with MEKK3. E14.5 mouse cortical lysates was immunoprecipitated with anti-MEKK3 antibody and probed for MEKK3 and WDR62. Underlying data can be found in S1 Data. BrdU, 5- bromo-2’-deoxyuridine; cKO, conditional knockout; CP, cortical plate; E, embryonic day; EGFP, enhanced green fluorescent protein; HA, influenza hemagglutinin; IgG, Immunoglobulin G; IP, immunoprecipitation; IZ, intermediate zone; KD, knockdown; MEKK3, mitogen-activated protein kinase kinase kinase 3; shRNA, short hairpin RNA; SVZ, subventricular zone; VZ, ventricular zone; WDR62, WD repeat domain 62; WT, wild-type.

WDR62 serves as a scaffold for the JNK pathway [34,35] and is critical for the maintenance of NPCs during brain development [40]. MAP3Ks (MKKKs) are important for development and tissue homeostasis and act as central regulators of cell fate during development [43]. To identify potential WDR62 interacting proteins, especially the MKKK that acts upstream of JNK and plays a role in neurogenesis, we screened for neurogenesis-disturbing MAP3Ks with different shRNAs via in utero electroporation at embryonic day 16.5 (E16.5) in rat (Fig 1A and S1 Fig) [40,44]. We found that only MEKK3 (also named MAP3K3) depletion with 3 different shRNAs incurred defects very similar to those resulting from WDR62 KD [40], including a dramatic reduction of NPCs in the proliferative regions of the ventricular and subventricular zones (VZ and SVZ) (Fig 1A). KD of MEKK2 or MEKK4 did not disturb the distribution of cells in a similar way as WDR62 KD (S1 Fig). In addition, we have shown previously that KO or KD of another MKKK, TAK1, only affects the migration of newborn neurons [45].

MEKK3 is a serine/threonine kinase that can be activated by different signaling pathways. Previous studies showed that MEKK3 is essential for T-cell or cancer cell proliferation [46,47]. The similar defects induced by depletion of MEKK3 and WDR62 suggest that MEKK3, like WDR62, may control NPC proliferation and differentiation. To test this, we crossed the *Mekk3*^*flox/flox*^ mice that we generated previously [46] with *Nestin-Cre* mice to obtain *Mekk3*^*flox/flox*^;*Nestin-Cre* conditional knockout (*Mekk3* cKO) mice in which *Mekk3* was deleted in the NPCs. We inspected E16.5 cortical slices with different progenitor cell markers including Pax6 and Sox2 (markers for radial glial cells or apical progenitor cells) and Tbr2 (a marker for intermediate or basal progenitor cells). The thickness of Pax6^+^, Sox2^+^, and Tbr2^+^ cortical layers was reduced significantly in *Mekk3* cKO mice, indicating a decrease in NPCs (Fig 1B–1D). In addition, the thinner Pax6^+^ and Sox2^+^ cell layers were accompanied by broader cortical staining for Tuj1 (a marker for immature neurons) and decreased numbers of cells positive for phosphor-histone H3 (P-H3, a marker for mitotic activity), respectively (Fig 1B and 1C). Furthermore, we examined the effect of *Mekk3* KO on cell-cycle exit index. Both *Mekk3* cKO and their wild-type (WT) littermates were labeled at E16.5 with 5-bromo-2’-deoxyuridine (BrdU) to track cells undergoing DNA synthesis. Twenty-four hour later, Ki67 (a marker for proliferating cells) and BrdU^+^ cells were inspected in brain slices. We observed a substantial decrease in Ki67^+^ and BrdU^+^ cells and a significant increase in cell-cycle exit index (cells that had incorporated BrdU but were Ki67^−^) (Fig 1E), indicating an overall decrease in cell proliferation. Finally, we analyzed cell death in the *Mekk3* cKO cortices and did not observe an apparent increase in cell death in the VZ/SVZ (S2 Fig). Taken together, these findings indicate that MEKK3 is required for NPC proliferation and differentiation during cortical development.

### MEKK3 interacts with and stabilizes WDR62

Because both MEKK3 and WDR62 are required for neurogenesis, we postulated that MEKK3 might interact with WDR62 to regulate JNK activity and neurogenesis. To test this hypothesis, constructs encoding MEKK3 and WDR62 were transfected into HEK293 cells individually or in combination, and reciprocal coimmunoprecipitation experiments revealed that MEKK3 interacts with WDR62 (Fig 1F). In addition, an anti-MEKK3 antiserum was able to pull down endogenous WDR62 from E14.5 mouse cortex (Fig 1G).

**Fig 2:**
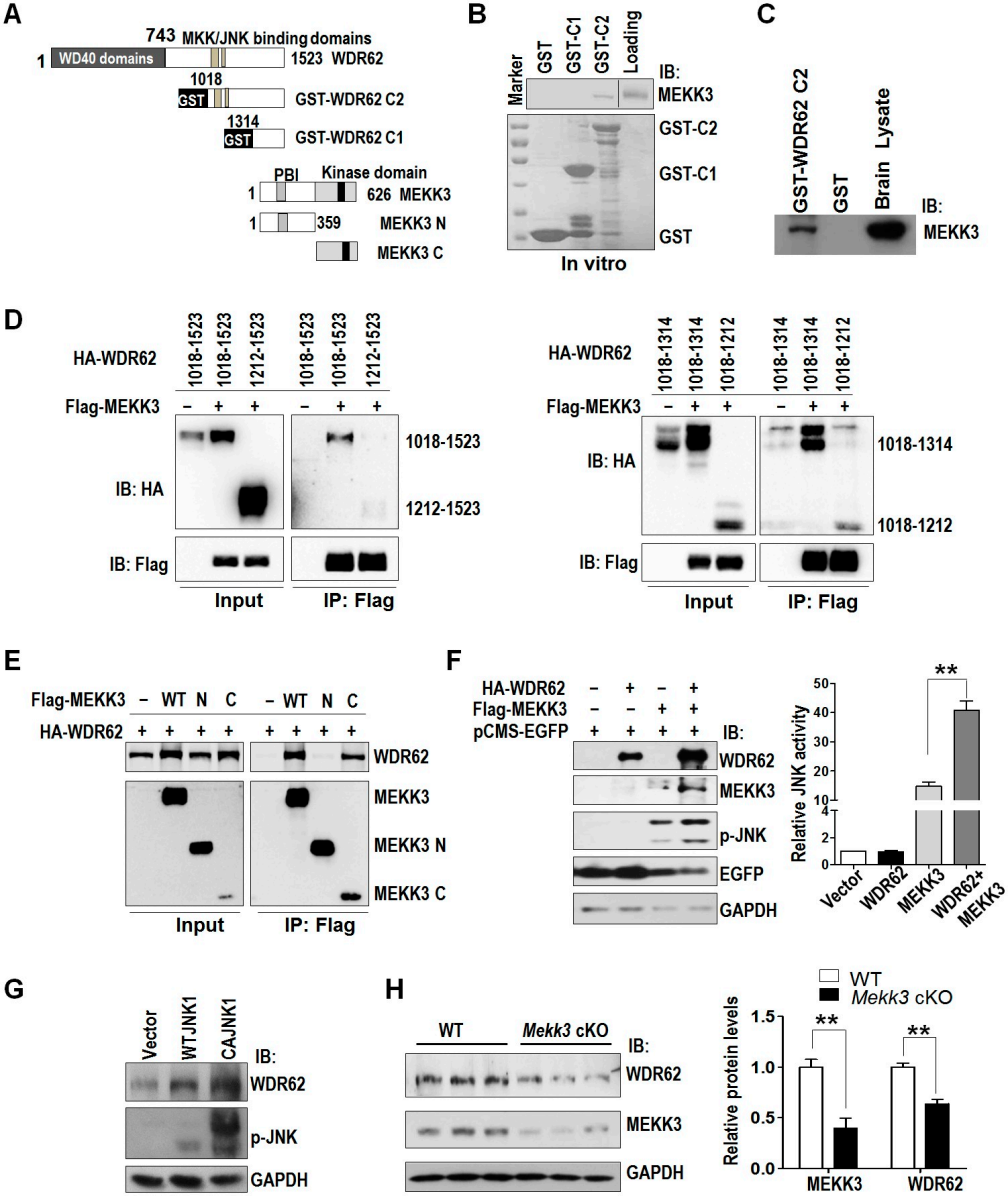
MEKK3 interacts with WDR62 and stabilizes WDR62. (A) Schematic representation of WDR62 and MEKK3 constructs. (B) MEKK3 interacts with WDR62 in vitro. In vitro–transcribed and translated MEKK3 was subjected to pull-down assay with purified GST-fused WDR62 fragments. (C) Purified GST fused C-terminal half of WDR62 pulls down MEKK3 from E14.5 mouse cortical lysate. (D) MEKK3 interacts with different fragments of WDR62. (E) The C-terminal half of MEKK3 interacts with WDR62. (F) Left panel: cells were transfected as indicated. Cell lysates were probed with HA, Flag, and p-JNK, with GAPDH serving as a loading control and GFP as transfection efficiency control. Right panel: quantification of left panel from 3 independent experiments. (G) Vector, WT JNK1, or CAJNK1 was transfected in 293 cells; 24 hours later, cells were collected and analyzed for WDR62 and JNK activity. (H) Left panel: E16.5 cortices from 3 *Mekk3* cKO and 3 WT littermates were analyzed for endogenous WDR62 and MEKK3 by western blot. Right panel: quantification of left panel. All data are means ± SEM; **P < 0.01. Underlying data can be found in S1 Data. CA, constitutively active; E, embryonic day; EGFP, enhanced green fluorescent protein; cKO, conditional knockout; GFP, green fluorescent protein; GST, glutathione-S-transferase; HA, influenza hemagglutinin; IP, immunoprecipitation; JNK, c-Jun N-terminal kinase; MEKK3, mitogen-activated protein kinase kinase kinase 3; WDR62, WD repeat domain 62; WT, wild-type.

We went on to generate different truncation mutants of WDR62 and MEKK3 in order to identify the potential binding site in MEKK3 and WDR62 (Fig 2A). Using the glutathione-S-transferase (GST)-fused WDR62 truncation mutant (WDR62 C2, aa1018-1523), which includes the MKK and JNK binding domains [34,35], we performed in vitro GST pull-down assays and noticed that MEKK3 could bind to WDR62 C2, but not the WDR62 C1 (aa1314-1523) mutant or GST (Fig 2B). GST pull-down experiment on E14.5 mouse brain lysate was able to pull down endogenous MEKK3 (Fig 2C). These results indicate that WDR62 and MEKK3 association is direct and mapped to 1018–1314 at the WDR62 C-terminal.

Because the WDR62 MKK4/7 binding domain was mapped to aa1212-84 [35], the MEKK3 binding domain is likely to be located within aa1018-1212 on WDR62 unless there is an overlap with the MKK4/7 binding domain. Therefore, we characterized in more detail the potential MEKK3 binding motif on WDR62. As shown in Fig 2D, MEKK3 could interact with aa1018-1523, 1018–1314, and 1018–1212 of WDR62. This indicates that the binding motif for MEKK3 in WDR62 is located within aa1018-1212.

The domain structure of MEKK3 consists of a conserved kinase domain and a PB1 domain in the C- and N-terminals, respectively (Fig 2A). Through reciprocal coimmunoprecipitation analysis, we detected that WDR62 interacted with the C-terminal half of MEKK3, but not the N-terminal half of MEKK3 (Fig 2E and S3A Fig). In order to investigate whether a synergistic effect exists between WDR62 and MEKK3, as what has been shown previously for POSH and the MLK family members [36,37], WDR62 and MEKK3 were expressed either alone or in combination in 293 cells. As shown in Fig 2F, when WDR62 and MEKK3 were coexpressed, the level of JNK activity (phosphorylated form of JNK) was significantly enhanced compared to WDR62 or MEKK3 expressed alone. Interestingly, the levels of WDR62 and MEKK3 protein were also much higher when coexpressed (Fig 2F). This suggests that WDR62 and MEKK3 play a synergistic role in the activation of JNK signaling, likely by mutual stabilization of the two proteins.

To determine whether JNK1 is also involved in the regulation of WDR62 levels, downstream of MEKK3, WT JNK1 and a constitutively active form of JNK1 (CA JNK1) were expressed in 293 cells. As shown in Fig 2G, the WDR62 protein level was higher in WT JNK1-expressed cells, and even higher in CA JNK1-expressing cells compared with vector controls, in accordance with the level of JNK activity. Importantly, endogenous WDR62 protein levels were much lower in E16.5 *Mekk3* cKO cortices (Fig 2H). However, KD or overexpression of MEKK3 had no significant effect on WDR62 mRNA levels (S3B and S3C Fig). Taken together, the above results indicate that MEKK3 and JNK1 regulate WDR62 expression at the post-transcriptional level.

### WDR62 controls neurogenesis and brain size through the regulation of JNK activity

**Fig 3:**
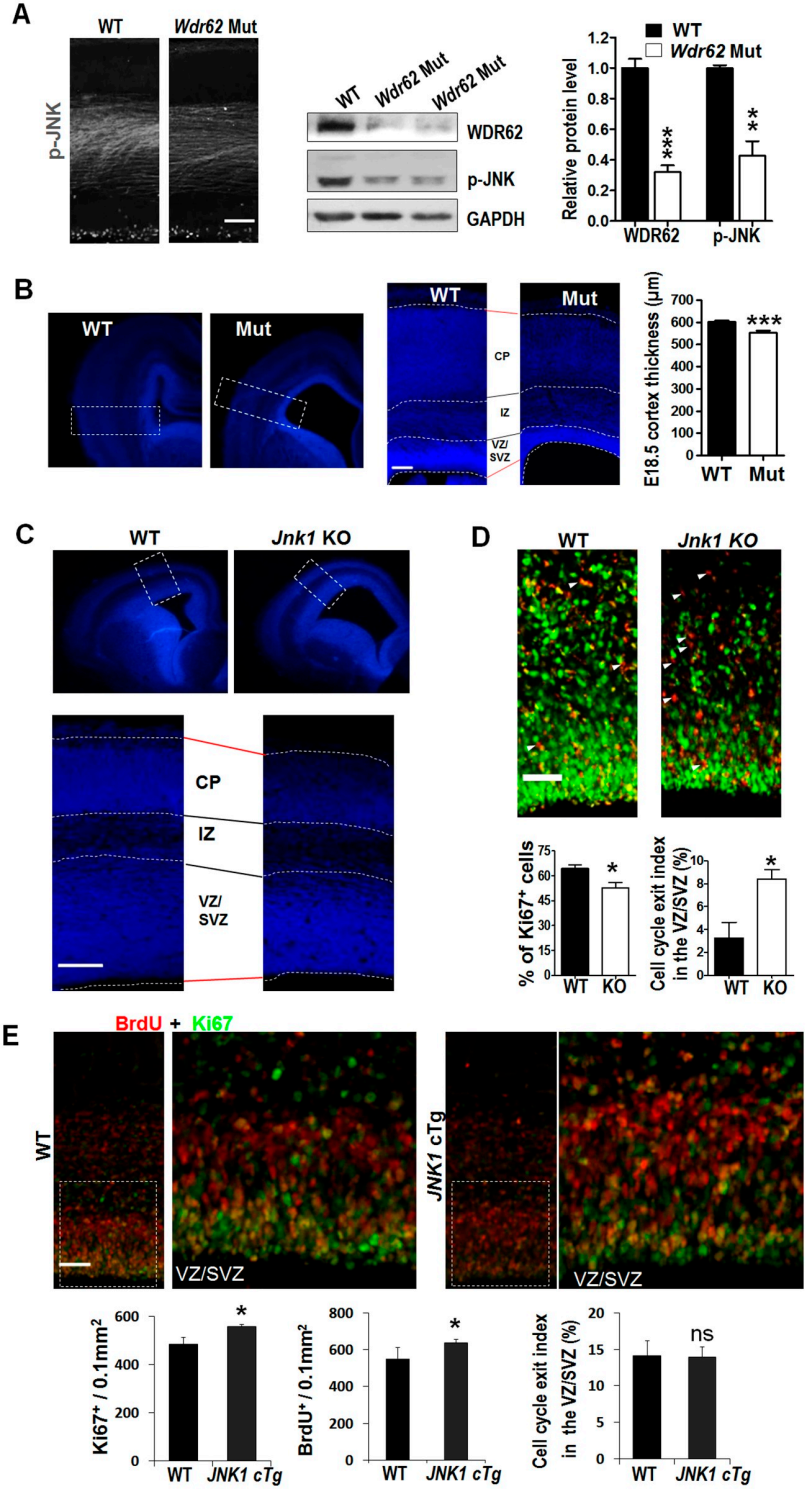
WDR62 controlled JNK activity is important for neurogenesis. (A) Left panel: coronal sections from E14.5 *Wdr62* mutant and WT littermates were stained with p-JNK antibody. Middle panel: Immunoblot analysis of WDR62 and p-JNK levels in E14.5 WT and mutant cortices. Right panel: quantification of p-JNK level from 2 independent experiments. WT *n* = 3, *wdr62* Mut *n* = 4. (B) Left panel: DAPI staining of coronal section of E18.5 *Wdr62* mutant and WT brains. Enlarged views of the cortical area are shown in the middle panel. Right panel: the average cortical thickness was measured at the position of the rectangle. WT *n* = 20 and *Wdr62* mutant *n* = 22 brain slices in 13 brains. (C) DAPI staining of coronal section of E16.5 *Jnk1* KO and WT brains. Enlarged views of the cortical area are shown in the lower panel. (D) Images of the VZ/SVZ of cortices from E16.5 *Jnk1* KO or WT littermates labeled with BrdU at E16 and stained for Ki67 and BrdU. Arrowheads mark BrdU^+^ but Ki67^−^ cells that have exited the cell cycle. Lower panels: quantification of Ki67^+^ cells and cell-cycle exit index. WT and *Jnk1* KO *n* = 3. (E) Images of the VZ/SVZ of cortices from E17.5 *JNK1* cTg or WT littermates labeled with BrdU at E16.5 and stained for Ki67 and BrdU. Lower panels: quantification of cell-cycle exit index and BrdU^+^ or Ki67^+^ cells. WT, *n* = 3; *Jnk1* cTg, *n* = 4. *n*: number of brain slices from different brains. Scale bars: 50 μm. All data are means ± SEM; ns *P* > 0.05, *P < 0.05, ***P < 0.001. Underlying data can be found in S1 Data. BrdU, 5-bromo-2’-deoxyuridine; CP, cortical plate; E, embryonic day; JNK1, c-Jun N-terminal kinase 1; ns, not significant; IZ, intermediate zone; KO, knockout; Mut, mutant; SVZ, subventricular zone; VZ, ventricular zone; WDR62, WD repeat domain 62; WT, wild-type.

*Jnk1* and *Jnk2* double-deficient mouse embryos develop exencephaly and die around E11–12 [48]. *Jnk1* KO induced pluripotent stem cells (iPSCs) are impaired in their ability to develop into neural precursors in vitro [49]. We have shown previously that JNK1 KD and WDR62 KD cause similar defects during cortical development [40]. We therefore generated a *Wdr62* null mutant [50] and investigated further the relationship between WDR62 and JNK activity during brain development. As shown in Fig 3A, the levels of JNK activity were significantly reduced in the *Wdr62* mutant cortex as indicated by immunostaining and western blotting, which is consistent with previous findings in different systems [50,51]. We went on to inspect whether KO of *Jnk1* would result in similar defects as KO of *Wdr62* during cortical development. As observed in *Wdr62* mutant mice (Fig 3B), *Jnk1* KO brains at E16 also showed enlarged ventricles and a thinner cortex, especially in the VZ/SVZ (Fig 3C). In addition, we observed a significant decrease in Ki67^+^ cells and an increase in cell-cycle exit index in *Jnk1* KO cortices (Fig 3D).

Because WDR62 regulates JNK activity, we postulated that WDR62 might regulate NPC proliferation and differentiation through JNK1. To test this hypothesis, we investigated whether the defects in *Wdr62* mutants can be rescued by JNK1. We first generated conditional transgenic mice expressing CA *JNK1* (S4 Fig). The transgenic mice were crossed with *Nestin-Cre* mice in order to express *CA JNK1* in NPCs (*JNK1* cTg, hereafter). As observed in cells, *CA JNK1* expression increased JNK activity and WDR62 protein level in the cortex (S5A and S5B Fig). We next examined the effect of JNK1 activation on NPC development through BrdU labeling. As shown in Fig 3E, *JNK1* cTg cortices showed a significant increase in BrdU^+^ and Ki67^+^ cells, while the cell-cycle exit index was comparable between *JNK1* cTg and their WT littermates.

**Fig 4:**
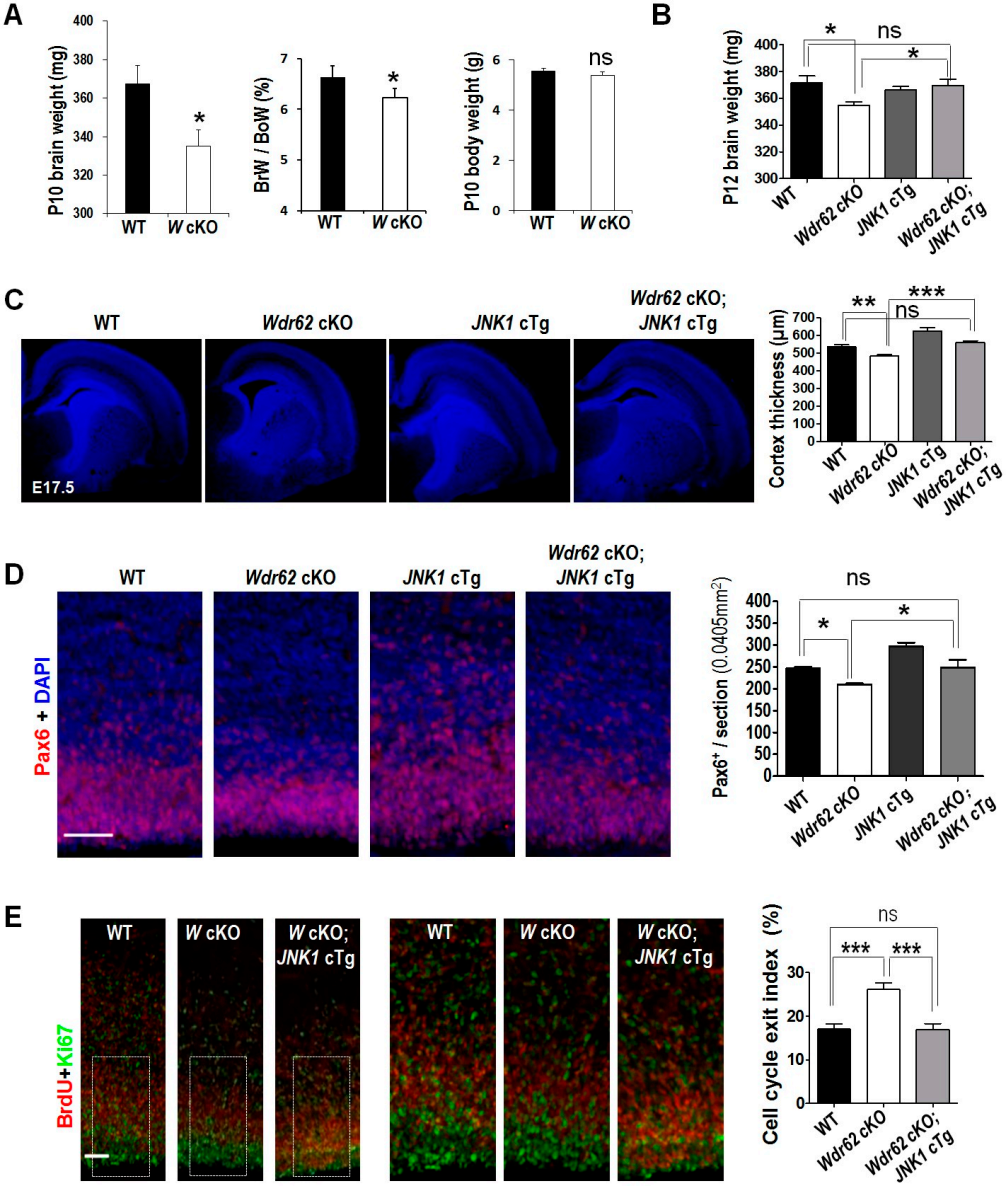
WDR62 regulates NPC proliferation, differentiation, and brain size through JNK1. (A) Brain and body weight of P10 *Wdr62*^*flox/flox*^;*Nestin-cre* (*Wdr62* cKO) and WT mice and their ratio of brain weight (BrW) to body weight (BoW). Four *Wdr62* cKO and WT littermates were analyzed. (B) Brain weight of P12 mice. *Wdr62* cKO, *n* = 16; *JNK1* cTg, *n* = 4; WT, *n* = 10 and *Wdr62* cKO;*JNK1* cTg, *n* = 10. (C) Coronal sections of E17.5 cortices stained with DAPI. Right panel: quantification of cortical thickness at the position of rectangle. WT *n* = 9; *Wdr62* cKO *n* = 14; *JNK1* cTg *n* = 7; *Wdr62* cKO;*JNK1* cTg *n* = 6. (D) Coronal sections from E17.5 cortices stained with Pax6 and DAPI. Right panel: quantification of Pax6^+^ cells per section. WT and *Wdr62* cKO *n* = 4; *JNK1* cTg and *Wdr62* cKO;*JNK1* cTg *n* = 3. (E) Images of cortices from E17.5 littermates labeled with BrdU at E16.5 and stained for Ki67 and BrdU. Right panel: quantification of cell-cycle exit index. WT *n* = 8; *Wdr62* cKO and *Wdr62* cKO/*JNK1* cTg *n* = 7. All Data are means ± SEM; ***P < 0.001; **P < 0.01; *P < 0.05; ns *P* > 0.05; *n*: number of brain slices from different brains. All scale bars: 50 μm. Underlying data can be found in S1 Data. BoW, body weight; BrdU, 5-bromo-2’-deoxyuridine; BrW, brain weight; cKO, conditional knockout; E, embryonic day; JNK, c-Jun N-terminal kinase; NPC, neural progenitor cell; ns, not significant; P12, postnatal day 12; WDR62, WD repeat domain 62; WT, wild-type.

Because *Wdr62* mutants were sterile [50], we used brain-specific *Wdr62*^*flox/flox*^*;Nestin-Cre* mice (*Wdr62* cKO) for further investigations. Similar to our *Wdr62* mutants, *Wdr62* cKOs showed reduced brain weight, enlarged ventricles, and a thinner cortex (Fig 4A–4C and S5C and S5D Fig). By crossing *Wdr62* cKO with *JNK1* cTg mice, we were able to generate *Wdr62* cKO, JNK1 cTg, and Wdr62 cKO;*JNK1* cTg genotypes. Compared with WT littermates, *Wdr62* cKO, but not *JNK1* cTg, mice had decreased brain weight at P12. The brain weight *of Wdr62* cKO;*JNK1* cTg mice was increased compared with *Wdr62* cKO mice, and comparable to that of controls (Fig 4B). This indicates that *Wdr62* cKO-induced microcephaly can be rescued by increased JNK1 activity. Similarly, *JNK1* cTg also rescued the reduced cortex thickness and enlarged lateral ventricle phenotypes in *Wdr62* cKO mice (Fig 4C and S5D Fig).

Because *Wdr62* deficiency leads to defects in NPC proliferation and differentiation, we investigated whether those defects could be rescued in *Wdr62* cKO;*JNK1* cTg mice. As shown in Fig 4D, the number of Pax6^+^ cells was significantly reduced in *Wdr62* cKOs and significantly increased in *JNK1 cTgs*, while *Wdr62* cKO;*JNK1* cTg double mutants were comparable to controls. Moreover, we observed a significant increase in cell-cycle exit in *Wdr62* cKOs but not in *Wdr62* cKO;*JNK1 cTg* cortices (Fig 4E). Taken together, these findings indicate that WDR62 regulates NPC proliferation and differentiation through JNK1.

### FBW7 negatively regulates WDR62 protein stability through the proteasomal pathway

**Fig 5:**
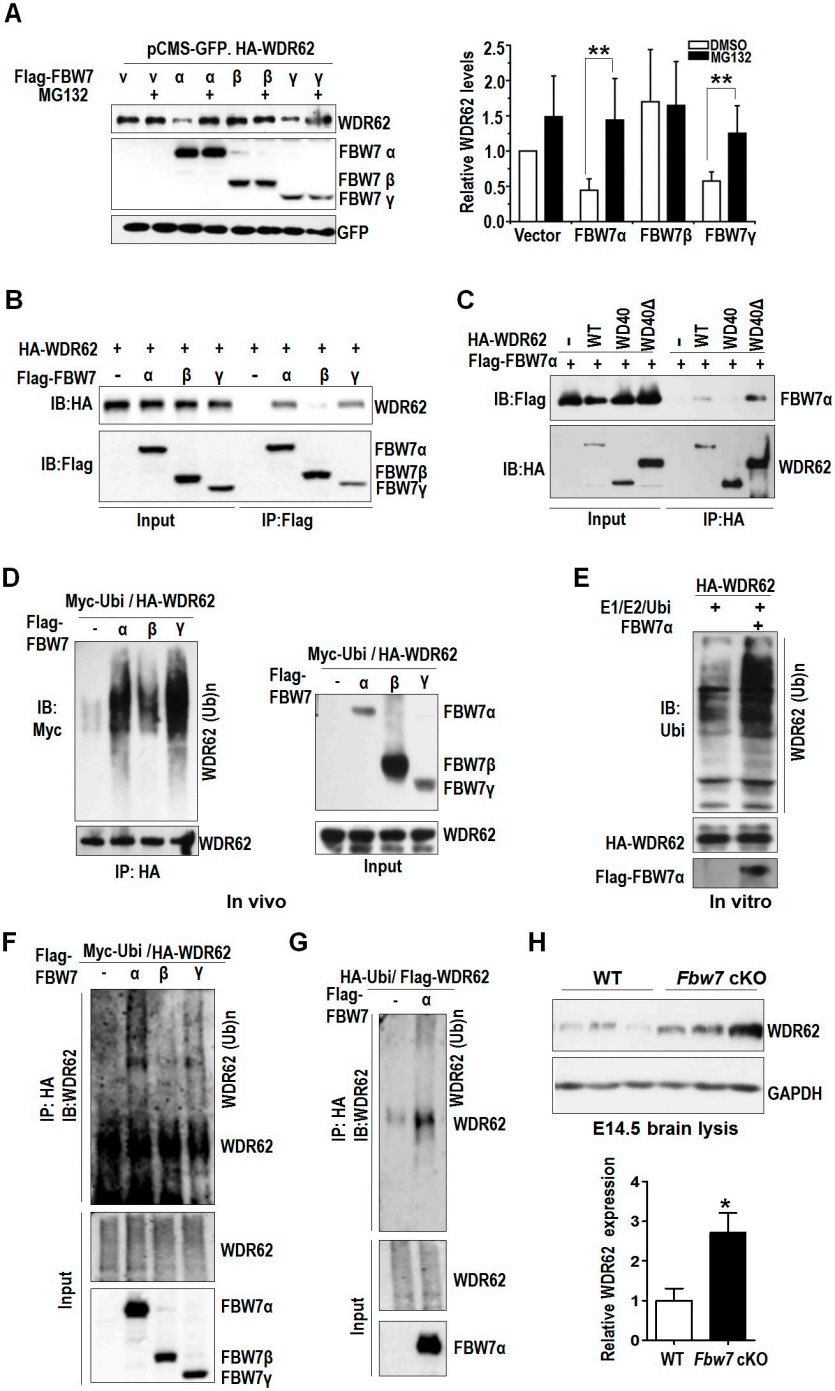
FBW7 regulates WDR62 protein stability through the proteasomal pathway. (A) Left panel: FBW7α and γ reduces WDR62 protein level. HEK293 cells were transfected as indicated; 16 hours later, cells were treated with MG132 for 4 hours and analyzed by immunoblotting with HA and Flag antibodies. Right panel: quantification of left panel in 3 independent experiments. Relative levels of WDR62 were normalized with GFP. (B) FBW7α and γ interacts with WDR62. Cells transfected as in indicated and were immunoprecipitated with Flag antibody and analyzed. (C) FBW7α interacts with the C-terminal half of WDR62 (WD40Δ). FBW7α was cotransfected with WDR62, WDR62 WD40 (N-terminal half of WDR62) and WDR62 WD40Δ. Cell lysates were immunoprecipitated with HA and analyzed. (D) FBW7α induces WDR62 ubiquitination in cells. HA-WDR62 and Myc-Ub were cotransfected with FBW7α, immunoprecipitated with HA antibody, and probed with Flag and Myc antibodies. (E) FBW7α induces WDR62 ubiquitination in vitro. HA-WDR62 or Flag-FBW7α were expressed in cells, purified by immunoprecipitation and then subjected to in vitro ubiquitination assay. (F) Upper smear signal by immunoblotting with WDR62 indicate stronger ubiquitination of FBW7α. (G) HA-ubi pulled out more WDR62 in WDR62 and FBW7α transfected lyses. (H) E14.5 cortices from 3 *Fbw7* cKO and 3 WT littermates were analyzed for endogenous WDR62 with GAPDH as control. All data are means ± SEM; *P < 0.05, **P < 0.01. Underlying data can be found in S1 Data. cKO, conditional knockout; E, embryonic day; FBW7, F-box and WD repeat domain-containing protein 7; GFP, green fluorescent protein; HA, influenza hemagglutinin; WDR62, WD repeat domain 62; WT, wild-type.

WDR62 and MEKK3 play a synergistic role in the activation of JNK. However, under physiological conditions, a negative regulatory mechanism likely exists to prevent cell death incurred by sustained JNK activation. We noticed that a JNK1-target phosphorylation site in WDR62, T1053 [52], is located within LPQ**T**PEQE, a potential binding motif for the E3 ubiquitin ligase substrate recognition component FBW7. FBW7 plays an opposite role to WDR62 during brain development, promoting rather than antagonizing NPC differentiation [53,54]. This led us to postulate that FBW7 may interact with WDR62 to regulate WDR62 protein stability through the proteasomal pathway. WDR62 was transfected into HEK293 cells either alone or together with the 3 different isoforms of FBW7, FBW7α, β, and γ. Interestingly, WDR62 protein levels appeared lower when coexpressed with FBW7 α or γ in particular, and the reduction could be significantly blocked by MG132 (Fig 5A). We therefore performed a coimmunoprecipitation analysis and detected an interaction of WDR62 with FBW7α and γ but not FBW7β (Fig 5B). In addition, FBW7α interacted with the C-terminal half of WDR62 (WD40Δ) but not the N-terminal half of WDR62 that consists primarily of WD40 domains (Fig 5C). To examine whether FBW7 possesses E3 ligase activity towards WDR62, we assessed WDR62 ubiquitination both in vivo and in vitro. Both FBW7α expression in cells (Fig 5D) and purified FBW7α in an in vitro assay (Fig 5E) induced the ubiquitination of WDR62. To rule out the possibility that the smeared ubiquitin signals were from WDR62-associated proteins, we performed the immunoprecipitation of influenza hemagglutinin (HA)-tagged WDR62, blotted for WDR62, and detected significantly increased upper smear signal when coexpressed with FBW7α and γ but not FBW7β (Fig 5F). Reciprocal immunoprecipitation for HA-ub also pulled out more WDR62 in FBW7α overexpressed cells (Fig 5G). Importantly, WDR62 protein levels were significantly higher in E14.5, E15.5, and E17.5 *Fbw7* cKO brains (Fig 5H and S6A and S6B Fig). However, deficiency of FBW7 had no significant effect on WDR62 mRNA level (S6B Fig). These results indicate that Fbw7 negatively regulates WDR62 protein stability through the proteasomal pathway.

Previous study indicates that FBW7 controls neural stem cell differentiation in midbrain [54]. We inspected the distribution of cells in cortex electroporated with Ctrl shRNA, *Fbw7* shRNA, *Wdr62* shRNA, and *Wdr62* shRNA with *Fbw7* shRNA. FBW7 KD led to reduced percentage of cells in the SVZ, intermediate zone (IZ), and cortical plate (CP), while WDR62 KD had the opposite phenotype. The phenotype was partially neutralized when *Fbw7* shRNA and *Wdr62* shRNA were cotransfected together (S6C and S6D Fig). This further supports the notion that WDR62 and FBW7 plays an opposite role in NPC development.

### JNK1 induced phosphorylation of WDR62 is important for the ubiquitination of WDR62 by FBW7

**Fig 6:**
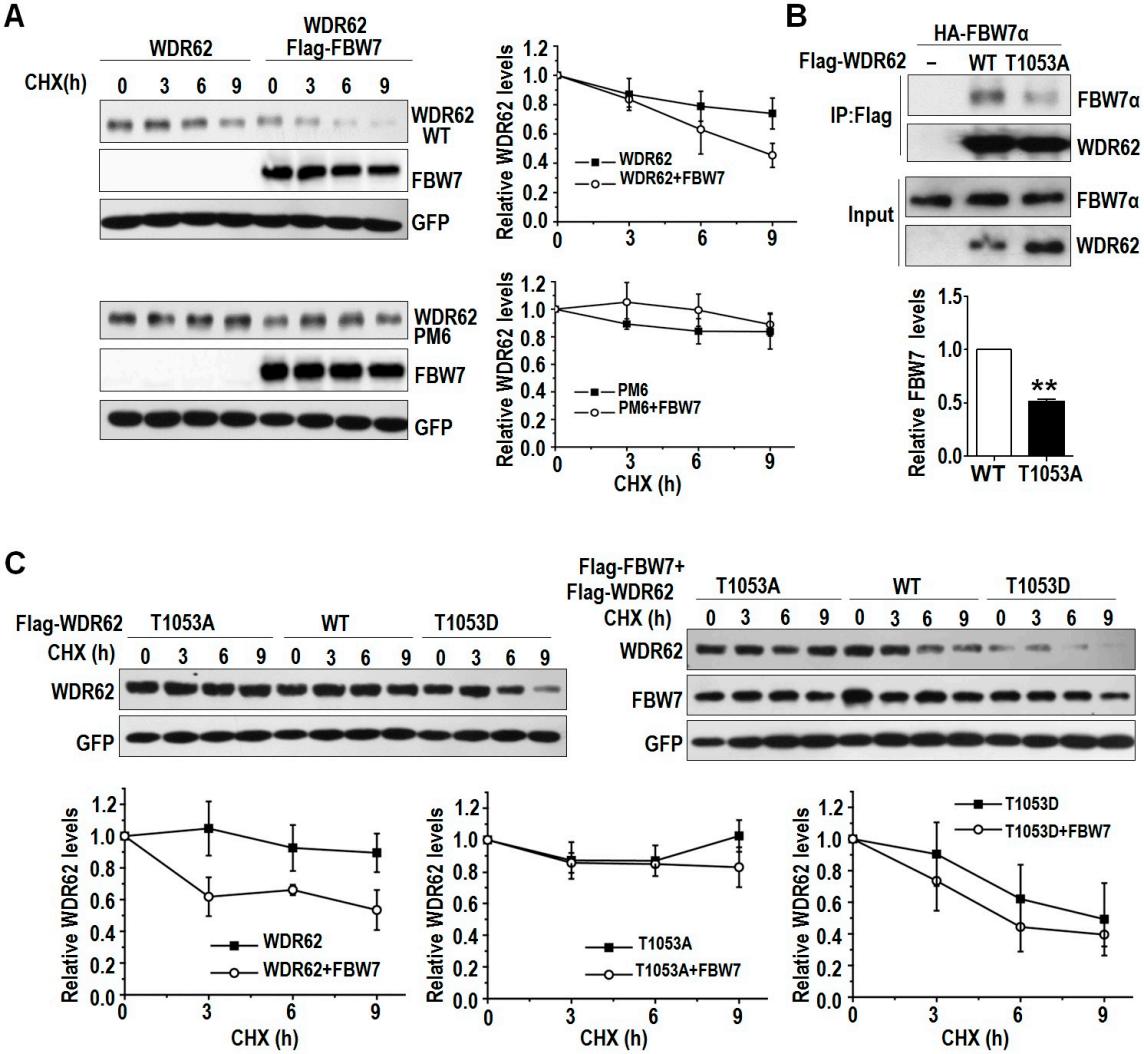
WDR62 T1053 phosphorylation impaired FBW7-mediated degradation. (A) Left: WT WDR62 or WDR62 PM6 was transfected independently or cotransfected with FBW7α and the half-life of WDR62 was examined by CHX chase assay as indicated. Right: quantification of (left) in 3 independent experiments. (B) The interaction between FBW7α and WDR62 1053A is much weaker. Lower: quantification of (upper) in 2 independent experiments. Relative levels of FBW7 (IP) were normalized with FBW7α (Input). (C) WT WDR62 or WDR62 T1053A and WDR62 T1053D were single transfected or cotransfected with FBW7α, and the half-life of WDR62 was examined by CHX chase assay as indicated. Lower panel: quantification of upper panel in 3 independent experiments. All data are means ± SEM. Underlying data can be found in S1 Data. CHX, cycloheximide; FBW7, F-box and WD repeat domain-containing protein 7; IP, immunoprecipitation; WDR62, WD repeat domain 62; WT, wild-type.

We went on to perform a cycloheximide (CHX) time course analysis and found that WDR62 was degraded more rapidly when coexpressed with FBW7α (Fig 6A). We also utilized a WDR62 mutant (PM6, L1299A/L1301A) that is unable to bind to and activate JNKs [55], and compared with WT WDR62, the PM6 mutant was very stable even when coexpressed with FBW7α (Fig 6A), suggesting that the interaction between JNK and WDR62 is important for the destabilization of WDR62.

**Fig 7:**
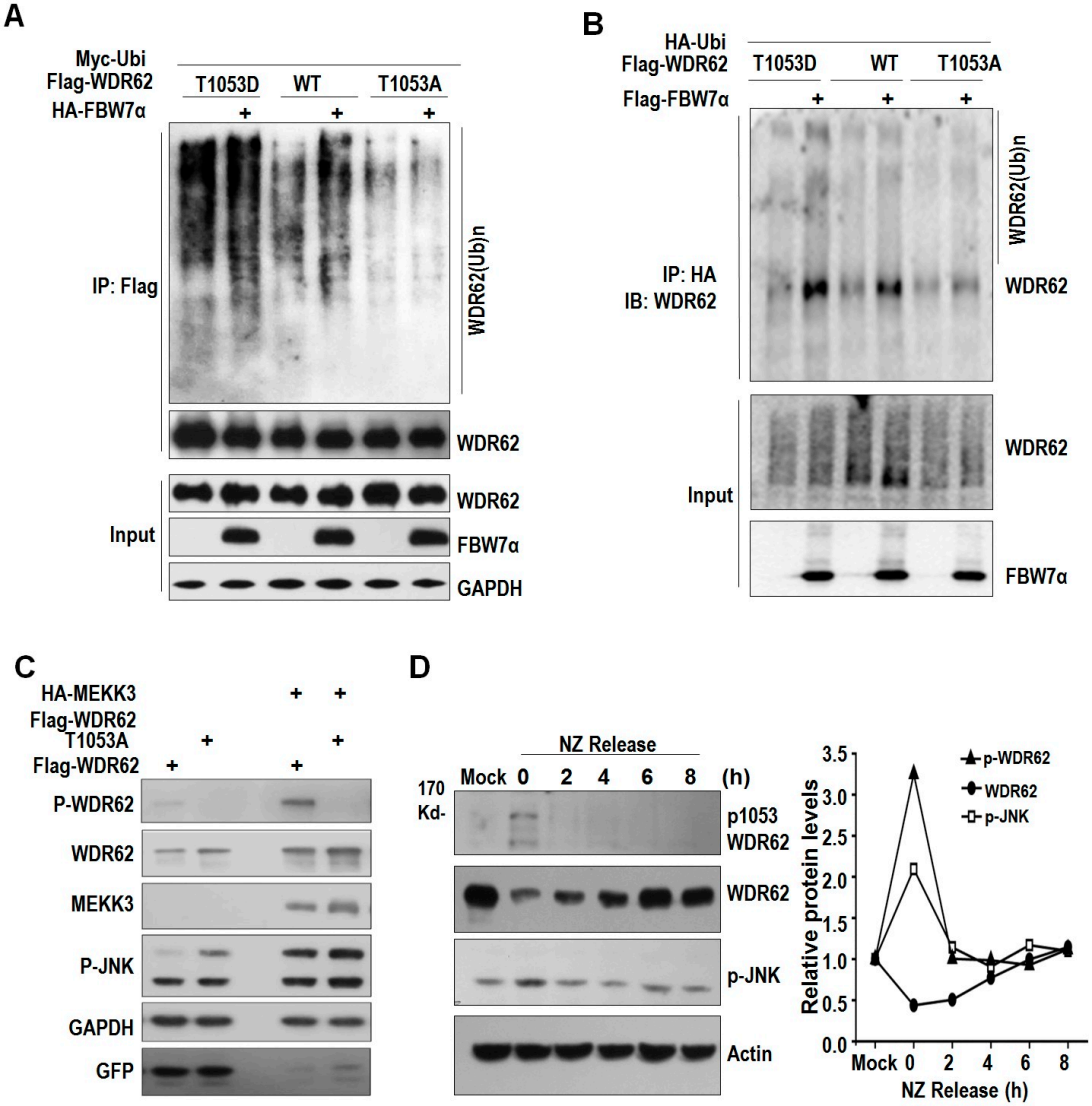
WDR62 T1053 phosphorylation is critical for its regulation by FBW7. (A) FBW7 induces more ubiquitination of WDR62 T1053D than WDR62 WT and WDR62 T1053A. (B) FBW7 mediated HA-ubi pull out more WDR62 in T1053D compared with WT and T1053A. (C) HEK293 cells were transfected with WT WDR62 or WDR62 T1053A alone or in combination with MEKK3. 24 hours later, cell lysates were probed with p-WDR62, WDR62, MEKK3, p-JNK, GAPDH, and GFP antibodies. (D) Endogenous WDR62 T1053 phosphorylation declines after exiting M phase (release from nocodazole arrest), is correlated with JNK activity but negatively correlated with WDR62 level. Underlying data can be found in S1 Data. FBW7, F-box and WD repeat domain-containing protein 7; GFP, green fluorescent protein; IP, immunoprecipitation; JNK, c-Jun N-terminal kinase; MEKK3, mitogen-activated protein kinase kinase kinase 3; NZ, nocodazole; WDR62, WD repeat domain 62; WT, wild-type.

Because JNK1 can induce the phosphorylation of WDR62 T1053 [52], we investigated the role of this modification in WDR62 stability. When WDR62 T1053 was mutated to alanine, its interaction with FBW7 decreased significantly compared with that of WT WDR62 (Fig 6B). We went on to make a phosphomimetic mutant, WDR62 T1053D, and found that it was less stable than WT WDR62, while WDR62 T1053A was more stable when cotransfected with FBW7α (Fig 6C and S7 Fig). We further examined the ubiquitination of WDR62 mutants induced by FBW7. When coexpressed with FBW7, an increased ubiquitination of WT WDR62 but not WDR62 T1053A was detected (Fig 7A and 7B). Consistently, the ubiquitination level of WDR62 T1053D was significantly higher than that of WT WDR62. WDR62 T1053A seemed to be more stable than WT WDR62 and could induce JNK activity more significantly when coexpressed with MEKK3 (Fig 7C).

JNK activity has been shown to increase during G2 and M phases of the cell cycle and decline after exiting M phase [56]. We arrested HeLa cells in anaphase with nocodazole and then released the cell-cycle arrest. As shown in Fig 7D, JNK activity declined after release from nocodazole arrest, as did the phosphorylation of endogenous WDR62 at T1053. Meanwhile, the level of WDR62 protein increased correspondingly. Taken together, our results indicate that the phosphorylation of WDR62 at T1053 by JNK is critical for its interaction with FBW7 and subsequent ubiquitination and degradation.

## Discussion

**Fig 8:**
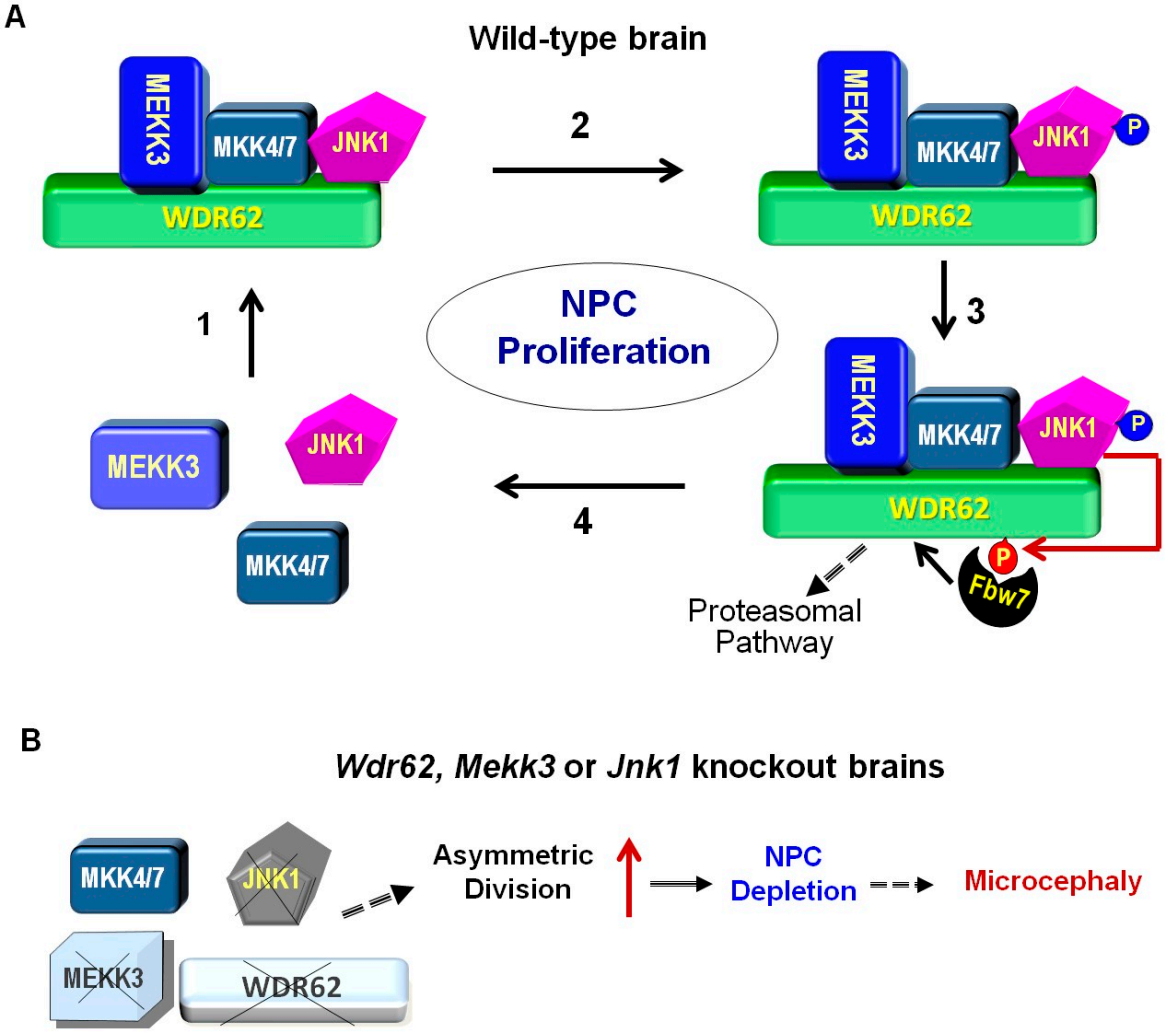
MEKK3 coordinates with FBW7 to regulate WDR62 stability and neurogenesis during brain development. (A) In the WT brain, the WDR62 scaffold organizes a protein complex including MEKK3, MKK4/7, and JNK1 to control NPC development during corticogenesis (1). The expression of WDR62 is positively regulated by MEKK3 which induced JNK activation (phosphorylation) (blue) (2). It is also negatively regulated subsequently by JNK-induced phosphorylation at T1053 (red) (3). The phosphorylation of WDR62 at T1053 recruits FBW7 to lead to the ubiquitination and degradation of WDR62 through the proteasomal pathway (3), and the down-regulation of JNK activity (4). (B) JNK1 cannot be activated properly in *Wdr62*, *Mekk3*, or *Jnk1* KO brains, leading to defects in NPC maintenance (possibly due to increase of asymmetric division), which may cause microcephaly. FBW7, F-box and WD repeat domain-containing protein 7; JNK1, c-Jun N-terminal kinase 1; KO, knockout; MEKK3, mitogen-activated protein kinase kinase kinase 3; MKK4/7, MAP kinase kinase 4/7; NPC, neural progenitor cell; WDR62, WD repeat domain 62; WT, wild-type.

Several MCPH proteins (ASPM, WDR62, CDK5RAP2, CEP63, CEP135, CEP152, CPAP, MCPH1, and STIL) have been shown to play a role in neurogenesis [7,57–60]. Our study reveals the mechanisms that regulate the stability of MCPH-associated protein WDR62 and NPC proliferation and differentiation during brain development. Specifically, we demonstrate that MEKK3 interacts with WDR62 to stabilize WDR62 and regulates JNK activity in a synergic way. On the other hand, JNK activity also regulates the phosphorylation of WDR62 at T1053 in a feedback loop which facilities the recruitment of FBW7 degradation of WDR62 (Fig 8). In addition, KO of MEKK3 or JNK1 phenocopies WDR62 KO in the dysregulation of NPC development. Transgenic expression of JNK1 can rescue the defects of WDR62, indicating a critical role of JNK signaling pathway in cell fate determination and NPC maintanence.

### MEKK3 cooperates with WDR62 in the regulation of JNK signaling and neurogenesis

Through a functional screen, we have found that MEKK3 KD induces very similar phenotypes as WDR62 KD [40], such as NPC depletion in the embryonic neocortex, suggesting defects in the maintenance of NPC proliferation and the occurrence of premature differentiation. This notion is supported by the significant decrease in cycling cells (Ki67^+^ and P-H3^+^) and an increase in cell-cycle exit index in the *Mekk3* cKO embryonic neocortex. Meanwhile, we observed a considerable reduction in Pax6-, Sox2-, and Tbr2-positive NPCs accompanied by an increase in Tuj1-positive immature neurons in these mice. Thus, our findings indicate a role for MEKK3 in the proliferation and differentiation of NPC during neurogenesis.

The similar function of MEKK3 and WDR62 led us to explore their relationship and confirm their interaction in the embryonic brain. Interestingly, WDR62 and MEKK3 are likely to play a synergistic role in the activation of JNK signaling as well as in the elevation of each other’s protein levels. In addition, expression of JNK1 elevated WDR62 levels, while endogenous levels of WDR62 were much lower in *Mekk3* cKO cortices. Therefore, we can postulate that MEKK3 regulates the protein level of WDR62 through JNK signaling.

### JNK signaling is important for cortical neurogenesis

Previous studies have shown that depletion or mutation of several MCPH proteins leads to the premature cell-cycle exit of NPCs and consequently to premature neuronal differentiation or cell death during neurogenesis [42,57,61,62]. Several MCPH proteins such as MCPH1 and ASPM have been shown to regulate the Chk1-Cdc25b and Wnt signaling pathways respectively to control brain size [62,63]. Our studies indicate that the JNK signaling plays a critical role in the normal function of WDR62. First, *Jnk1* KO mice have phenotypes very similar to our *Wdr62* KO and cKO mice, including premature differentiation of NPCs, enlarged lateral ventricles, and thinner cortices during cortical development. These defects in *Wdr62* cKO mice can be largely rescued by the transgenic expression of CA-JNK1. In addition, mice with deletion of kinases upstream of JNK1 have phenotypes somewhat similar to *Wdr62* and *Jnk1* mutants. For example, brain-specific *Mkk7 o*r *Mkk4* KO mice display either enlarged embryonic brain ventricles or reduced brain size [64]. Taken together, all these studies imply that WDR62 cooperates with MEKK3, MKKs, and JNK1 in the regulation of brain development.

### FBW7 negatively regulates WDR62 stability through the proteasomal pathway

The E3 ligase FBW7 is important for normal brain development, and KO of *Fbw7* inhibits NPC differentiation [54], the opposite defect of that caused by *Wdr62* KO. We have confirmed the interaction between WDR62 and FBW7. Interestingly, WDR62 T1053, which is phosphorylated by JNK1 and localizes within the FBW7 binding motif of WDR62, is important for the interaction between WDR62 and FBW7. In addition, phosphorylation of T1053 is crucial for the regulation of WDR62 stability by FBW7, through ubiquitination and degradation of WDR62.

Previous studies have shown that WDR62 protein level is cell-cycle dependent [22,52]. However, the underlying mechanism is unknown. Two mutants—WDR62 L1299A/L1301A, which cannot bind to JNK, and WDR62 T1053A—are more stable than WT WDR62, indicating the involvement of JNK signaling in the regulation WDR62 expression. JNK activity increases during G2 and M phases of cell cycle [56]. Intriguingly, the level of WDR62 phosphorylated at T1053 declines after cells are released from nocodazole arrest (M anaphase). This is accompanied by the decline in JNK activity and the elevation of WDR62 level, indicating that phosphorylation of T1053 is negatively correlated with WDR62 stability. We would like to propose a model (Fig 8) that JNK activation at G2/M phase leads to WDR62 phosphorylation at T1053, which will recruit FBW7 to induce the ubiquitination and degradation of WDR62. As the cell cycle progresses, JNK activity declines, and newly synthesized WDR62 will accumulate. Through the interaction with MEKK3, WDR62 is stabilized and promotes activation of JNK at G2/M phase. Thus, JNK-induced phosphorylation of T1053 is also likely to play a critical role in recruiting FBW7 and the degradation of WDR62 during cell-cycle progression. How MEKK3 and JNK1 stabilize WDR62 and activate JNK needs to be explored in the future.

Taken together, our results support a model in which the scaffold protein WDR62 organizes a protein complex that includes MEKK3, MKKs, and JNK1 to control the proliferation and differentiation of NPCs during corticogenesis (Fig 8). The expression of WDR62 is fine-tuned both positively by MEKK3 and JNK activity and negatively by JNK-induced phosphorylation of WDR62 at T1053. Thus, the coordinated reciprocal and bidirectional regulation among WDR62, MEKK3, JNK1, and FBW7 fine-tunes JNK signaling to control the balance between proliferation and differentiation of NPCs and prevent superfluous cell death incurred by sustained JNK activation during brain development.

## Materials and methods

### Ethics statement

All animal procedures used in this study were performed according to protocols approved by the Institutional Animal Care and Use Committee at the Institute of Genetics and Developmental Biology (IGDB), Chinese Academy of Sciences (CAS) (protocol number: AP2016053).

### Antibodies

The antibodies used for western blotting (for human) were as follows: GFP (abcam, ab290, 1:2,000), α-tubulin (CST, 3873s, 1:2,000), GAPDH (CST, 2118s, 1:2,000), Flag (MBL, M185, 1:2,000), Myc (MBL, M047-3, 1:5,000), HA (MBL, M180-3, 1:5,000), Phospho-JNK (CST, 9255, 1:1,000), and WDR62 (bethyl, A310-550A, 1:1,000). For mouse, they were as follows: WDR62 (abcam, 1:1,000), WDR62 antibody generated by MBL company using antigen VGQGGNQPKAGPLRAGTC, Phospho-WDR62 1053T (present from Dominic) [52], and MEKK3 (CST, 5727, 1:1,000). The antibodies used for immunostaining were Sox2 (abcam, ab97959, 1:1,000), Pax6 (Covance, PRB-278P, 1:400), Tbr2 (Millipore, ab2283, 1:1,000), β-III Tubulin/Tuj1 (abcam, ab7751, 1:1,000), γ-Tubulin (abcam, ab11316, 1:1,000), α-Tubulin (CST, 3873, 1:2,000), Phosph-Histone 3 (P-H3) (abcam, ab10543, 1:1,000), Nestin (abcam, ab6142, 1:1,000), GFP (abcam, ab13970, 1:1,000), Ki67 (abcam, ab15580, 1:1,000), BrdU (abcam, ab6326, 1:500), Phospho-JNK (abcam, ab124956, 1:1,000), activated-caspase3 (abcam, ab13847, 1:1,000). Nuclei were stained with DAPI (4’,6-diamidino-2-phenylindole) (Invitrogen).

### Plasmids

Human WDR62 WT, WD40 and WD40Δ truncations, or WDR62 mutants were created by cloning WDR62 cDNA sequence into the pCMS.EGFP (modified) (Flag) or pCDNA3.1-HA vector. Full-length MEKK3, MEKK3 N- and MEKK3 C-terminal truncations were cloned into the pCMV-Tag2B vector. Full-length MEKK3 was also cloned into the pCMS.EGFP (modified) (Flag) or pCDNA3.1-HA vector. For GST pull-down assay, full-length WDR62, WDR62 C2 (1018-1523aa), or WDR62 C1 (1314-1523aa) were cloned into pGEX6p1. Flag-FBW7α, β, and γ in pCDNA3.1 were kindly provided by Dr. Clurman [65]. All constructs were verified by sequencing.

### Generation of KO and transgenic mice

#### *Wdr62* cKO mice

*Wdr62*^+/flox^ mouse and *Wdr62* mutant (*Wdr62*^−/−^) mice were generated as described previously [50]. *Wdr62* cKO (*Wdr62*^*flox/flox*^*; Nestin-Cre*) mice were obtained by crossing *Nestin–Cre* mice with *Wdr62*^*flox/flox*^ mice.

#### *CA-JNK1* conditional transgenic mice

An HA-tagged human *JNKK2-JNK1* fusion construct was inserted into the pENTR1A vector (Life Technologies) as described in S4A Fig. To guarantee controlled and efficient monosite insertion of *JNKK2-JNK1* into the ubiquitously expressed ROSA26 locus, we used the Gateway Entry system (Life Technologies) (S4B Fig) [66]. Mice carrying the conditional floxed (loxP-STOP-loxP; LSL) LSL-*CA*-*JNK1* f/f allele were crossed with *Nestin-Cre* mice to generate mice expressing *CA-JNK1* in NPCs (*JNK1* cTg).

#### *Wdr62* cKO;*JNK1* cTg mice

By crossing *Wdr62* cKO with *JNK1* cTg mice, we were able to get *Wdr62* cKO, *JNK1 cTg*, and *Wdr62* cKO;*JNK1* cTg mice.

#### *Mekk3* cKO

Mice carrying the conditional (floxed, *Mekk3*^f/f^) allele [46] were crossed with *Nestin-Cre* mice to generate mice with a specific deletion in NPC (*Mekk3* cKO). *Jnk1* heterozygous (Jnk1+/−) KO mice (stock# 003553) were obtained from the Jackson Laboratory.

The day when a plug was observed in a female mouse was designated E0.5, and the day of birth was termed postnatal day 0 (P0). Mouse genotypes were determined by PCR. For all experiments, only littermate mice from the same breeding were used.

### Histology and immunofluorescence staining

For cryosections, tissues were fixed in 4% PFA, cryoprotected in 30% sucrose, and frozen in tissue freezing medium (TFM). Sections (thickness of 20–50 μm) were used for immunofluorescence staining. Immunofluorescence staining was carried out essentially as described previously [44,67].

### BrdU labeling

For single-pulse BrdU labeling, pregnant mice at defined pregnancy stages were injected intraperitoneally with 50 mg/g body weight of BrdU (Sigma-Aldrich) and were euthanized 12 to 24 hours after injection.

### Cell culture, transfection, immunoprecipitation, and western blotting

HEK293 cell culture, transfection, immunoprecipitation, and western blotting were performed as previously described [36]. Plasmids were transfected into HEK293 cells with VigoFect (VIGOROUS). For western blot of Figs 5F, 5G and 7B and S7 Fig, cells were treated with 20 μm MG132 for 4 hours before lyses (MG132 added). Densitometric analysis was performed using Image J software. The relative Integrated Density of western blot band was measured.

### In utero electroporation

In utero electroporation was performed as previously described [67]. Pregnant Sprague Dawley rats were provided by the animal center of IGDB. Rat *Mekk3* shRNA vector containing the following target sequences was used: shM1: 5’-GCCTTAGGATACTACTGTTA-3’; shM2: 5’-GCAGCAACATGATTGTGCA-3’; and shM3, 5’-GATCACAAAGACTACAATGA-3’. The human *MEKK3* shRNA target sequence was 5’-GCAGAGTGACGTCAGAATC-3’. The rat *Mekk2* shRNA target sequence was as follows: shRNA1: 5’-GAGCGAATTGTTCAGTATTA-3’; shRNA2: 5’-GAAGCAATGGCTGCCATCT-3’; and shRNA3: 5’-GCTGGATCCATTGTCTTTA-3’. The rat *Mekk4* shRNA target sequence was as follows: shRNA1: 5’-GAGGAAGCTGGATCCAAATG-3’; shRNA2: 5’-GAGTATCATAAAGAAGTTG-3’; and shRNA3: 5’-GCCTTTATTTCAGCTTTAC-3’. The rat *Fbw7* shRNA target sequence was 5’-CCTTCTCTGGAGAGAGAAA-3’.

### Statistical analysis

Sections were imaged on an LSM 700 (Carl Zeiss) confocal microscope as described [40]. Cell counts were analyzed with Imaris X64 or ImageJ. All data were analyzed using Excel and Prism software (Graph Pad Software, La Jolla, CA). Tests used were unpaired *t* test or one-way ANOVA paired with Tukey post-test.

## Supporting information

S1 Data Individual numerical values that underlie data displayed in Figs 1A–1E, 2F, 2H, 3A–3E, 4A–4E, 5A, 5H, 6A–6C and 7D, and S1B, S3B, S3C, S5B–S5D, S6B and S6D Figs. (XLSX)

S1 Fig MEKK2 and MEKK4 KD do not show the same phenotype as MEKK3 KD. (A) Coronal sections of rat cortices electroporated in utero with bicistronic constructs encoding both EGFP and MEKK2, MEKK4 shRNAs or control shRNA (Ctrl) at E16.5 and inspected at E20.5. (B) Quantification of cell distribution of EGFP^+^ cells. Data are means ± SEM; *P < 0.05. Ctrl, *n* = 8; *Mekk2* sh1, *n* = 5; *Mekk2* sh2, *n* = 7; *Mekk2* sh3, *n* = 10; *Mekk4* sh1, *n* = 5; *Mekk4* sh3, *n* = 6. *n*: brain slices from more than 3 independent brains. Scale bar: 50 μm. (C) KD efficiency of *Mekk2/Mekk4* shRNAs. HA-MEKK2, MEKK4 cDNAs were cotransfected with shCtrl or shRNAs into HEK293 cells; 48 h later, cell lysates were analyzed by immunoblotting with anti-HA antibody, with GAPDH/Tubulin serving as a loading control and GFP as a transfection efficiency control. Underlying data can be found in S1 Data. Ctrl, control; E, embryonic day; EGFP, enhanced green fluorescent protein; HA, influenza hemagglutinin; KD, knockdown; MEKK2, mitogen-activated protein kinase kinase kinase 2; MEKK4, mitogen-activated protein kinase kinase kinase 4; SC, scramble; shRNA, short hairpin RNA.
(TIF)

S2 Fig Images of E17.5 brain slices from WT and *Mekk3* cKO mice stained for the activated form of caspase 3 (green) and DAPI (blue). Scale bar: 50 μm. cKO, conditional knockout; E, embryonic day; MEKK3, mitogen-activated protein kinase kinase kinase 3; WT, wild-type.
(TIF)

S3 Fig MEKK3 interact with WDR62 and does not affect the mRNA levels of WDR62. (A) Reciprocal immunoprecipitation of Fig 2E. (B) Relative *WDR62* mRNA expression in *MEKK3* KD cells. HEK293 cells were transfected with scramble control or human *MEKK3* shRNA; 48 hours later, cells were collected for qPCR analysis. (C) Relative endogenous *WDR6*2 mRNA expression in human *MEKK3* overexpression cells. HEK293 cells were transfected with vector or HA-human *MEKK3*; 24 hours later, cells were collected for qPCR analysis. Underlying data can be found in S1 Data. HA, influenza hemagglutinin; IP, immunoprecipitation; MEKK3, mitogen-activated protein kinase kinase kinase 3; qPCR, quantitative PCR; shRNA, short hairpin RNA; WDR62, WD repeat domain 62.
(TIF)

S4 Fig Generation of JNK1 conditional transgenic mice. (A) Schematic of the JNKK2-JNK1 fusion constructs. The construct consists of a 3xHA tag, the human JNKK2 cDNA, (Gly-Gly) 5 repeats and the human JNK1 cDNA. The JNKK2-JNK1 fusion construct was inserted into the multiple cloning site of the pENTR1A vector (Life Technologies). (B) Targeting of the genomic ROSA26 locus with the JNKK2-JNK1 vector. In the pEntry clone, the JNKK2-JNK1 construct is flanked by lambda phage integrase recognition sites (*att*L) and thus can be efficiently inserted into the targeting vector carrying the corresponding heterotypic sites (*att*R). The targeting construct consists of a 5’-ROSA26 homology arm, a splice acceptor (SA) site, a PGK-neo-STOP cassette flanked by loxP-site (LSL), the JNKK2-JNK1 fusion construct, an IRES-eGFP reporter gene, a 3’-ROSA26 homology arm, and a PGK-DTA selection cassette. Screening PCR was performed using a forward primer indicated by arrow 1 and reverse primer indicated by arrow 2 in the 5’ region of the targeting construct. After Cre-mediated recombination, the LSL cassette is excised, and the JNKK2-JNK1 fusion construct is expressed in the genomic ROSA26 locus. For genotyping PCR, primers indicated by arrows 3, 4 located in the eGFP reporter gene were used. Arrowheads indicate loxP-sites. Underlying data can be found in S1 Data. eGFP, enhanced green fluorescent protein; HA, influenza hemagglutinin; IRES, internal ribosome entry site; JNK, c-Jun N-terminal kinase.
(TIF)

S5 Fig (A) Western blot analysis of JNK1 expression in the WT, *JNK1* cTg, *Wdr62* cKO, and *Wdr62*cKO;*JNK1* cTg brains at E14.5. GAPDH was used as a loading control. (B) Western blot analysis of WDR62 expression in the E16.5 WT and *JNK1* cTg mice brain. Right panels: quantification of WDR62 protein and mRNA expression. WT, *n* = 3; *JNK1* cTg, *n* = 2. (C) Body and brain weight of P3 *Wdr62*^*flox/flox*^;*Nestin-cre* (*Wdr62* cKO) and WT mice. Three *Wdr62* cKO and WT littermates were analyzed. (D) Quantification of ventricle area as a percentage of whole telencephalon area. WT, *n* = 10; *Wdr62* cKO, *n* = 14; *JNK1* cTg, *n* = 7; *Wdr62* cKO*;JNK1* cTg, *n* = 6. *n*: brain numbers. All data are means ± SEM; ***P < 0.001, *P < 0.05, ns *P* > 0.05. Underlying data can be found in S1 Data. cKO, conditional knockout; E, embryonic day; JNK1, Jun N-terminal kinase 1; ns, not significant; WDR62, WD repeat domain 62; WT, wild-type.
(TIF)

S6 Fig FBW7 regulates WDR62 stability at protein level. (A) E17.5 or E15.5 cortices from *Fbw7* cKO and WT littermates were analyzed by western blot for endogenous WDR62 with GAPDH as control. (B) Left panel: quantification of WDR62 protein levels compared to WT control in panel A. Middle and right panel: relative *Fbw7* and *Wdr62 mRNA* expression in 3 *Fbw7* cKO and 5 WT mice. (C) Coronal sections of rat cortices electroporated in utero with bicistronic constructs encoding both EGFP and *Wdr62* shRNA, *Fbw7* shRNA or control shRNA (Ctrl) at E16.5 and inspected at E20.5. Scale bar 50 μm. In E20.5 cortex: “ML” indicates the mantle layer, including the cortical SVZ, IZ, and CP. (D) Relative quantity of cells in VZ and ML in panel C. Scramble, *n* = 6; *Wdr62* shRNA1 (*Wdr62*sh1), *n* = 7; *Fbw7* shRNA1 (*Fbw7* sh1), *fbw7* sh1*; wdr62* sh1, *n* = 8. All data are means ± SEM; ***P < 0.001, **P < 0.01, *P < 0.05, ns *P* > 0.05. Underlying data can be found in S1 Data. CP, cortical plate; E, embryonic day; FBW7, F-box and WD repeat domain-containing protein 7; IZ, intermediate zone; ML, mantle layer; ns, not significant; SVZ, subventricular zone; VZ, ventricular zone; WDR62, WD repeat domain 62; WT, wild-type.
(TIF)

S7 Fig WDR62 T1053 is critical for FBW7-mediated degradation. WDR62 T1053A showed weak interaction with FBW7 compared with WDR62 WT. HEK293 cells were transfected with Flag-WDR62 and Flag-WDR62-T1053A either alone or in combination with HA-FBW7α; 16 hours later, cells were treated with MG132 for 4 hours. Cell lysates were immunoprecipitated with HA antibody and probed with HA or WDR62 antibodies. FBW7, F-box and WD repeat domain-containing protein 7; HA, influenza hemagglutinin; WDR62, IP, immunoprecipitation; WD repeat domain 62.
(TIF)

## Abbreviations

ASPM: abnormal spindle-like microcephaly-associated
BrdU: 5-bromo-2’-deoxyuridine
CA: constitutively active
CAS: Chinese Academy of Sciences
CHX: cycloheximide
cKO: conditional knockout
CP: cortical plate
E: embryonic day
EGFP: enhanced green fluorescent protein
FBW7: F-box and WD repeat domain-containing protein 7
GFP: green fluorescent protein
GST: glutathione-S-transferase
HA: influenza hemagglutinin
IGDB: Institute of Genetics and Developmental Biology
IP: immunoprecipitation
IPSC: induced pluripotent stem cell
IZ: intermediate zone
JIP: JNK-interacting protein
JNK: c-Jun N-terminal kinase
KD: knockdown
KO: knockout
MAPKKK: mitogen-activated protein kinase kinase kinase
MCPH: autosomal recessive primary microcephaly
MEKK2: mitogen-activated protein kinase kinase kinase 2
MEKK3: mitogen-activated protein kinase kinase kinase 3
MEKK4: mitogen-activated protein kinase kinase kinase 4
MKK: mitogen-activated protein kinase kinase
MKK4/7: MAP kinase kinase 4/7
NPC: neural progenitor cell
P: postnatal day
POSH: Plenty of SH3
shRNA: short hairpin RNA
SVZ: subventricular zone
VZ: ventricular zone
WDR62: WD repeat domain 62
WT: wild-type

## References

[1] Hu WF, Chahrour MH, Walsh CA. The diverse genetic landscape of neurodevelopmental disorders. Annu Rev Genomics Hum Genet. 2014;15:195–213. 10.1146/annurev-genom-090413-025600. . 25184530 PMC10591257

[2] Gotz M, Huttner WB. The cell biology of neurogenesis. Nat Rev Mol Cell Biol. 2005;6(10):777–88. 10.1038/nrm1739. . 16314867

[3] Breunig JJ, Haydar TF, Rakic P. Neural stem cells: historical perspective and future prospects. Neuron. 2011;70(4):614–25. 10.1016/j.neuron.2011.05.005. . 21609820 PMC3225274

[4] Kriegstein A, Alvarez-Buylla A. The glial nature of embryonic and adult neural stem cells. Annu Rev Neurosci. 2009;32:149–84. 10.1146/annurev.neuro.051508.135600. . 19555289 PMC3086722

[5] Kaindl AM, Passemard S, Kumar P, Kraemer N, Issa L, Zwirner A, et al Many roads lead to primary autosomal recessive microcephaly. Prog Neurobiol. 2010;90(3):363–83. 10.1016/j.pneurobio.2009.11.002. . 19931588

[6] Manzini MC, Walsh CA. What disorders of cortical development tell us about the cortex: one plus one does not always make two. Curr Opin Genet Dev. 2011;21(3):333–9. 10.1016/j.gde.2011.01.006. . 21288712 PMC3139684

[7] Thornton GK, Woods CG. Primary microcephaly: do all roads lead to Rome? Trends in Genetics. 2009;25(11):501–10. 10.1016/j.tig.2009.09.011. . 19850369 PMC2816178

[8] Yang YJ, Baltus AE, Mathew RS, Murphy EA, Evrony GD, Gonzalez DM, et al Microcephaly gene links trithorax and REST/NRSF to control neural stem cell proliferation and differentiation. Cell. 2012;151(5):1097–112. 10.1016/j.cell.2012.10.043. . 23178126 PMC3567437

[9] Knoblich JA. Mechanisms of asymmetric stem cell division. Cell. 2008;132(4):583–97. 10.1016/j.cell.2008.02.007. . 18295577

[10] Wang X, Lui JH, Kriegstein AR. Orienting fate: spatial regulation of neurogenic divisions. Neuron. 2011;72(2):191–3. 10.1016/j.neuron.2011.10.003. . 22017981 PMC3220621

[11] Franco SJ, Muller U. Shaping Our Minds: Stem and Progenitor Cell Diversity in the Mammalian Neocortex. Neuron. 2013;77(1):19–34. 10.1016/j.neuron.2012.12.022. . 23312513 PMC3557841

[12] Huttner WB, Kosodo Y. Symmetric versus asymmetric cell division during neurogenesis in the developing vertebrate central nervous system. Curr Opin Cell Biol. 2005;17(6):648–57. 10.1016/j.ceb.2005.10.005. . 16243506

[13] Hu DJ, Baffet AD, Nayak T, Akhmanova A, Doye V, Vallee RB. Dynein recruitment to nuclear pores activates apical nuclear migration and mitotic entry in brain progenitor cells. Cell. 2013;154(6):1300–13. 10.1016/j.cell.2013.08.024. . 24034252 PMC3822917

[14] Miyata T, Kawaguchi D, Kawaguchi A, Gotoh Y. Mechanisms that regulate the number of neurons during mouse neocortical development. Curr Opin Neurobiol. 2010;20(1):22–8. 10.1016/j.conb.2010.01.001. . 20138502

[15] Shen Q, Wang Y, Dimos JT, Fasano CA, Phoenix TN, Lemischka IR, et al The timing of cortical neurogenesis is encoded within lineages of individual progenitor cells. Nat Neurosci. 2006;9(6):743–51. 10.1038/nn1694. . 16680166

[16] Miller FD, Gauthier AS. Timing is everything: making neurons versus glia in the developing cortex. Neuron. 2007;54(3):357–69. 10.1016/j.neuron.2007.04.019. . 17481390

[17] Dehay C, Kennedy H. Cell-cycle control and cortical development. Nat Rev Neurosci. 2007;8(6):438–50. 10.1038/nrn2097. . 17514197

[18] Paridaen JT, Huttner WB. Neurogenesis during development of the vertebrate central nervous system. EMBO Rep. 2014;15(4):351–64. 10.1002/embr.201438447. . 24639559 PMC3989667

[19] He S, Nakada D, Morrison SJ. Mechanisms of stem cell self-renewal. Annu Rev Cell Dev Biol. 2009;25:377–406. 10.1146/annurev.cellbio.042308.113248. . 19575646

[20] Gilmore EC, Walsh CA. Genetic causes of microcephaly and lessons for neuronal development. Wiley Interdiscip Rev Dev Biol. 2013;2(4):461–78. 10.1002/wdev.89. . 24014418 PMC3767923

[21] Woods CG, Basto R. Microcephaly. Curr Biol. 2014;24(23):R1109–11. 10.1016/j.cub.2014.09.063. . 25465325

[22] Nicholas AK, Khurshid M, Desir J, Carvalho OP, Cox JJ, Thornton G, et al WDR62 is associated with the spindle pole and is mutated in human microcephaly. Nat Genet. 2010;42(11):1010–4. 10.1038/ng.682. . 20890279 PMC5605390

[23] Yu TW, Mochida GH, Tischfield DJ, Sgaier SK, Flores-Sarnat L, Sergi CM, et al Mutations in WDR62, encoding a centrosome-associated protein, cause microcephaly with simplified gyri and abnormal cortical architecture. Nat Genet. 2010;42(11):1015–20. 10.1038/ng.683. . 20890278 PMC2969850

[24] Bilguvar K, Ozturk AK, Louvi A, Kwan KY, Choi M, Tatli B, et al Whole-exome sequencing identifies recessive WDR62 mutations in severe brain malformations. Nature. 2010;467(7312):207–10. 10.1038/nature09327. . 20729831 PMC3129007

[25] Wollnik B. A common mechanism for microcephaly. Nat Genet. 2010;42(11):923–4. 10.1038/ng1110-923. . 20980985

[26] Khan MA, Rupp VM, Orpinell M, Hussain MS, Altmuller J, Steinmetz MO, et al A missense mutation in the PISA domain of HsSAS-6 causes autosomal recessive primary microcephaly in a large consanguineous Pakistani family. Hum Mol Genet. 2014;23(22):5940–9. 10.1093/hmg/ddu318. . 24951542

[27] Insolera R, Bazzi H, Shao W, Anderson KV, Shi SH. Cortical neurogenesis in the absence of centrioles. Nat Neurosci. 2014;17(11):1528–35. 10.1038/nn.3831. . 25282615 PMC4213237

[28] Martin CA, Ahmad I, Klingseisen A, Hussain MS, Bicknell LS, Leitch A, et al Mutations in PLK4, encoding a master regulator of centriole biogenesis, cause microcephaly, growth failure and retinopathy. Nat Genet. 2014 10.1038/ng.3122. . 25344692 PMC4676084

[29] Mirzaa GM, Vitre B, Carpenter G, Abramowicz I, Gleeson JG, Paciorkowski AR, et al Mutations in CENPE define a novel kinetochore-centromeric mechanism for microcephalic primordial dwarfism. Hum Genet. 2014;133(8):1023–39. 10.1007/s00439-014-1443-3. . 24748105 PMC4415612

[30] Shohayeb B, Lim NR, Ho U, Xu Z, Dottori M, Quinn L, et al The Role of WD40-Repeat Protein 62 (MCPH2) in Brain Growth: Diverse Molecular and Cellular Mechanisms Required for Cortical Development. Mol Neurobiol. 2017 10.1007/s12035-017-0778-x. . 28940170

[31] Kadir R, Harel T, Markus B, Perez Y, Bakhrat A, Cohen I, et al ALFY-Controlled DVL3 Autophagy Regulates Wnt Signaling, Determining Human Brain Size. PLoS Genet. 2016;12(3):e1005919 10.1371/journal.pgen.1005919. . 27008544 PMC4805177

[32] Moawia A, Shaheen R, Rasool S, Waseem SS, Ewida N, Budde B, et al Mutations of KIF14 cause primary microcephaly by impairing cytokinesis. Ann Neurol. 2017;82(4):562–77. 10.1002/ana.25044. . 28892560

[33] Cabello OA, Eliseeva E, He WG, Youssoufian H, Plon SE, Brinkley BR, et al Cell cycle-dependent expression and nucleolar localization of hCAP-H. Mol Biol Cell. 2001;12(11):3527–37. 10.1091/mbc.12.11.3527. . 11694586 PMC60273

[34] Wasserman T, Katsenelson K, Daniliuc S, Hasin T, Choder M, Aronheim A. A novel c-Jun N-terminal kinase (JNK)-binding protein WDR62 is recruited to stress granules and mediates a nonclassical JNK activation. Mol Biol Cell. 2010;21(1):117–30. 10.1091/mbc.E09-06-0512. . 19910486 PMC2801705

[35] Cohen-Katsenelson K, Wasserman T, Khateb S, Whitmarsh AJ, Aronheim A. Docking interactions of the JNK scaffold protein WDR62. Biochem J. 2011;439(3):381–90. 10.1042/BJ20110284. . 21749326 PMC3462610

[36] Xu ZH, Kukekov NV, Greene LA. POSH acts as a scaffold for a multiprotein complex that mediates JNK activation in apoptosis. EMBO J. 2003;22(2):252–61. 10.1093/emboj/cdg021. . 12514131 PMC140096

[37] Xu Z, Kukekov NV, Greene LA. Regulation of apoptotic c-Jun N-terminal kinase signaling by a stabilization-based feed-forward loop. Mol Cell Biol. 2005;25(22):9949–59. 10.1128/MCB.25.22.9949-9959.2005. . 16260609 PMC1280282

[38] Kukekov NV, Xu Z, Greene LA. Direct interaction of the molecular scaffolds POSH and JIP is required for apoptotic activation of JNKs. J Biol Chem. 2006;281(22):15517–24. 10.1074/jbc.M601056200. . 16571722

[39] Davis RJ. Signal transduction by the JNK group of MAP kinases. Cell. 2000;103(2):239–52. . 11057897 10.1016/s0092-8674(00)00116-1

[40] Xu D, Zhang F, Wang Y, Sun Y, Xu Z. Microcephaly-associated protein WDR62 regulates neurogenesis through JNK1 in the developing neocortex. Cell Rep. 2014;6(1):104–16. 10.1016/j.celrep.2013.12.016. . 24388750

[41] Chen JF, Zhang Y, Wilde J, Hansen KC, Lai F, Niswander L. Microcephaly disease gene Wdr62 regulates mitotic progression of embryonic neural stem cells and brain size. Nat Commun. 2014;5:3885 10.1038/ncomms4885. . 24875059 PMC4216695

[42] Jayaraman D, Kodani A, Gonzalez DM, Mancias JD, Mochida GH, Vagnoni C, et al Microcephaly Proteins Wdr62 and Aspm Define a Mother Centriole Complex Regulating Centriole Biogenesis, Apical Complex, and Cell Fate. Neuron. 2016;92(4):813–28. 10.1016/j.neuron.2016.09.056. . 27974163 PMC5199216

[43] Craig EA, Stevens MV, Vaillancourt RR, Camenisch TD. MAP3Ks as Central Regulators of Cell Fate During Development. Dev Dyn. 2008;237(11):3102–14. 10.1002/Dvdy.21750. . 18855897

[44] Zhang F, Xu D, Yuan L, Sun Y, Xu Z. Epigenetic regulation of Atrophin1 by lysine-specific demethylase 1 is required for cortical progenitor maintenance. Nat Commun. 2014;5:5815 10.1038/ncomms6815. . 25519973 PMC4284801

[45] Zhang F, Yu J, Yang T, Xu D, Chi Z, Xia Y, et al A Novel c-Jun N-terminal Kinase (JNK) Signaling Complex Involved in Neuronal Migration during Brain Development. J Biol Chem. 2016;291(22):11466–75. 10.1074/jbc.M116.716811. . 27026702 PMC4882418

[46] Wang XF, Chang X, Facchinetti V, Zhuang Y, Su B. MEKK3 Is Essential for Lymphopenia-Induced T Cell Proliferation and Survival. J Immunol. 2009;182(6):3597–608. 10.4049/jimmunol.0803738. . 19265138 PMC2923428

[47] Santoro R, Zanotto M, Carbone C, Piro G, Tortora G, Melisi D. MEKK3 Sustains EMT and Stemness in Pancreatic Cancer by Regulating YAP and TAZ Transcriptional Activity. Anticancer Res. 2018;38(4):1937–46. 10.21873/anticanres.12431. . 29599309

[48] Kuan CY, Yang DD, Samanta Roy DR, Davis RJ, Rakic P, Flavell RA. The Jnk1 and Jnk2 protein kinases are required for regional specific apoptosis during early brain development. Neuron. 1999;22(4):667–76. . 10230788 10.1016/s0896-6273(00)80727-8

[49] Zhang Q, Mao J, Zhang X, Fu H, Xia S, Yin Z, et al Role of Jnk1 in development of neural precursors revealed by iPSC modeling. Oncotarget. 2016;7(38):60919–28. 10.18632/oncotarget.11377. . 27556303 PMC5308626

[50] Zhou Y, Qin Y, Qin YY, Xu BY, Guo T, Ke HN, et al Wdr62 is involved in meiotic initiation via activating JNK signaling and associated with POI in humans. PLoS Genet. 2018;14(8). 10.1371/journal.pgen.1007463. . 30102701 PMC6107287

[51] Prinz E, Aviram S, Aronheim A. WDR62 mediates TNFalpha dependent JNK activation via TRAF2-MLK3 axis. Mol Biol Cell. 2018:mbcE17080504. 10.1091/mbc.E17-08-0504. . 30091641 PMC6233063

[52] Lim NR, Yeap YY, Zhao TT, Yip YY, Wong SC, Xu D, et al Opposing roles for JNK and Aurora A in regulating the association of WDR62 with spindle microtubules. J Cell Sci. 2015;128(3):527–40. 10.1242/jcs.157537. . 25501809 PMC4311131

[53] Matsumoto A, Onoyama I, Sunabori T, Kageyama R, Okano H, Nakayama KI. Fbxw7-dependent Degradation of Notch Is Required for Control of "Stemness" and Neuronal-Glial Differentiation in Neural Stem Cells. J Biol Chem. 2011;286(15):13754–64. 10.1074/jbc.M110.194936. . 21349854 PMC3075719

[54] Hoeck JD, Jandke A, Blake SM, Nye E, Spencer-Dene B, Brandner S, et al Fbw7 controls neural stem cell differentiation and progenitor apoptosis via Notch and c-Jun. Nat Neurosci. 2010;13(11):1365–72. 10.1038/nn.2644. . 20935640

[55] Bogoyevitch MA, Yeap YYC, Qu ZD, Ngoei KR, Yip YY, Zhao TT, et al WD40-repeat protein 62 is a JNK-phosphorylated spindle pole protein required for spindle maintenance and timely mitotic progression. J Cell Sci. 2012;125(21):5096–109. 10.1242/jcs.107326. . 22899712 PMC3533392

[56] Gutierrez GJ, Tsuji T, Chen MF, Jiang W, Ronai ZA. Interplay between Cdh1 and JNK activity during the cell cycle. Nature Cell Biology. 2010;12(7):686–U130. 10.1038/ncb2071. 20581839 PMC2899685

[57] Fish JL, Kosodo Y, Enard W, Paabo S, Huttner WB. Aspm specifically maintains symmetric proliferative divisions of neuroepithelial cells. Proc Natl Acad Sci U S A. 2006;103(27):10438–43. 10.1073/pnas.0604066103. . 16798874 PMC1502476

[58] Lizarraga SB, Margossian SP, Harris MH, Campagna DR, Han AP, Blevins S, et al Cdk5rap2 regulates centrosome function and chromosome segregation in neuronal progenitors. Development. 2010;137(11):1907–17. 10.1242/dev.040410. . 20460369 PMC2867323

[59] Manzini MC, Walsh CA. What disorders of cortical development tell us about the cortex: one plus one does not always make two. Curr Opin Genet Dev. 2011;21(3):333–9. 10.1016/j.gde.2011.01.006. 21288712 PMC3139684

[60] Wollnik B. A common mechanism for microcephaly. Nat Genet. 2010;42(11):923–4. 10.1038/ng1110-923. . 20980985

[61] Buchman JJ, Tseng HC, Zhou Y, Frank CL, Xie Z, Tsai LH. Cdk5rap2 interacts with pericentrin to maintain the neural progenitor pool in the developing neocortex. Neuron. 2010;66(3):386–402. 10.1016/j.neuron.2010.03.036. . 20471352

[62] Gruber R, Zhou ZW, Sukchev M, Joerss T, Frappart PO, Wang ZQ. MCPH1 regulates the neuroprogenitor division mode by coupling the centrosomal cycle with mitotic entry through the Chk1-Cdc25 pathway. Nat Cell Biol. 2011;13(11):1325–U100. 10.1038/ncb2342. . 21947081

[63] Buchman JJ, Durak O, Tsai LH. ASPM regulates Wnt signaling pathway activity in the developing brain. Genes Dev. 2011;25(18):1909–14. 10.1101/gad.16830211. . 21937711 PMC3185963

[64] Yamasaki T, Kawasaki H, Arakawa S, Shimizu K, Shimizu S, Reiner O, et al Stress-activated protein kinase MKK7 regulates axon elongation in the developing cerebral cortex. J Neurosci. 2011;31(46):16872–83. 10.1523/JNEUROSCI.1111-11.2011. . 22090513 PMC6633308

[65] Koepp DM, Schaefer LK, Ye X, Keyomarsi K, Chu C, Harper JW, et al Phosphorylation-dependent ubiquitination of cyclin E by the SCFFbw7 ubiquitin ligase. Science. 2001;294(5540):173–7. 10.1126/science.1065203. . 11533444

[66] Nyabi O, Naessens M, Haigh K, Gembarska A, Goossens S, Maetens M, et al Efficient mouse transgenesis using Gateway-compatible ROSA26 locus targeting vectors and F1 hybrid ES cells. Nucleic Acids Research. 2009;37(7). 10.1093/nar/gkp112 PMC2673446 19279185

[67] Sun Y, Fei T, Yang T, Zhang F, Chen YG, Li H, et al The suppression of CRMP2 expression by bone morphogenetic protein (BMP)-SMAD gradient signaling controls multiple stages of neuronal development. J Biol Chem. 2010;285(50):39039–50. 10.1074/jbc.M110.168351. . 20926379 PMC2998125

