# Supplementary figures and images for: MEKK3 coordinates with FBW7 to regulate WDR62 stability and neurogenesis
Source: PLoS Biol. 2018 Dec 19;16(12):e2006613. doi: 10.1371/journal.pbio.2006613 (PMC6347294; doi:10.1371/journal.pbio.2006613)

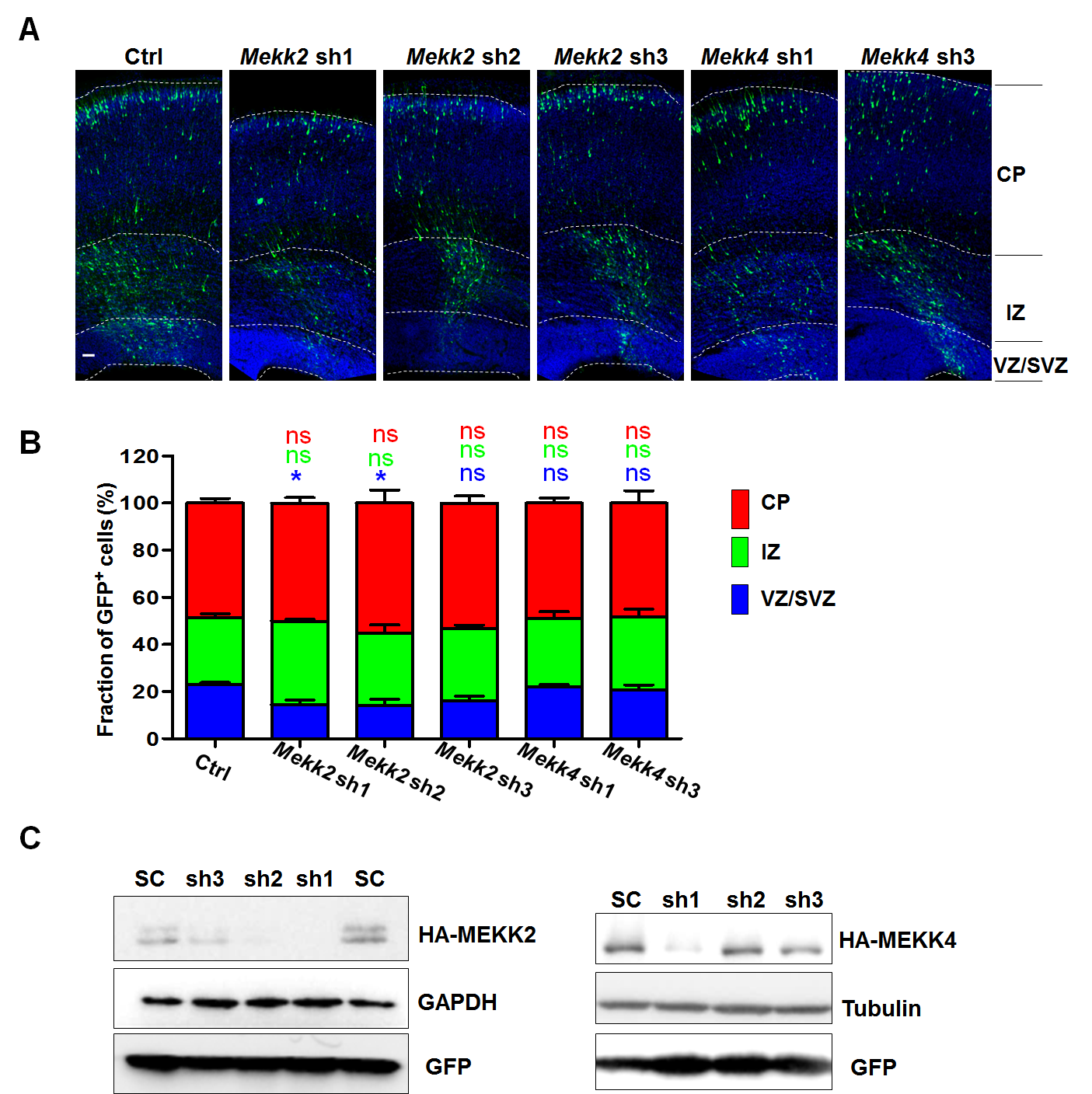

Supplement: S1 Fig — (A) Coronal sections of rat cortices electroporated in utero with bicistronic constructs encoding both EGFP and MEKK2, MEKK4 shRNAs or control shRNA (Ctrl) at E16.5 and inspected at E20.5. (B) Quantification of cell distribution of EGFP+ cells. Data are means ± SEM; *P < 0.05. Ctrl, n = 8; Mekk2 sh1, n = 5; Mekk2 sh2, n = 7; Mekk2 sh3, n = 10; Mekk4 sh1, n = 5; Mekk4 sh3, n = 6. n: brain slices from more than 3 independent brains. Scale bar: 50 μm. (C) KD efficiency of Mekk2/Mekk4 shRNAs. HA-MEKK2, MEKK4 cDNAs were cotransfected with shCtrl or shRNAs into HEK293 cells; 48 h later, cell lysates were analyzed by immunoblotting with anti-HA antibody, with GAPDH/Tubulin serving as a loading control and GFP as a transfection efficiency control. Underlying data can be found in S1 Data. Ctrl, control; E, embryonic day; EGFP, enhanced green fluorescent protein; HA, influenza hemagglutinin; KD, knockdown; MEKK2, mitogen-activated protein kinase kinase kinase 2; MEKK4, mitogen-activated protein kinase kinase kinase 4; SC, scramble; shRNA, short hairpin RNA. (TIF) [file pbio.2006613.s002.tif]

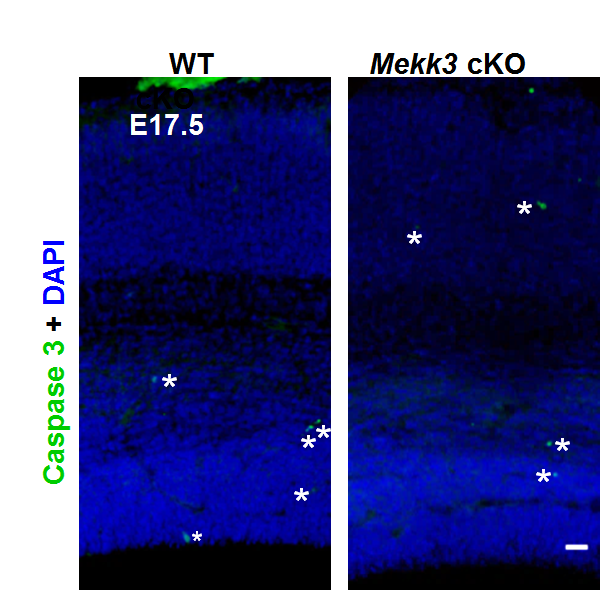

Supplement: S2 Fig — Scale bar: 50 μm. cKO, conditional knockout; E, embryonic day; MEKK3, mitogen-activated protein kinase kinase kinase 3; WT, wild-type. (TIF) [file pbio.2006613.s003.tif]

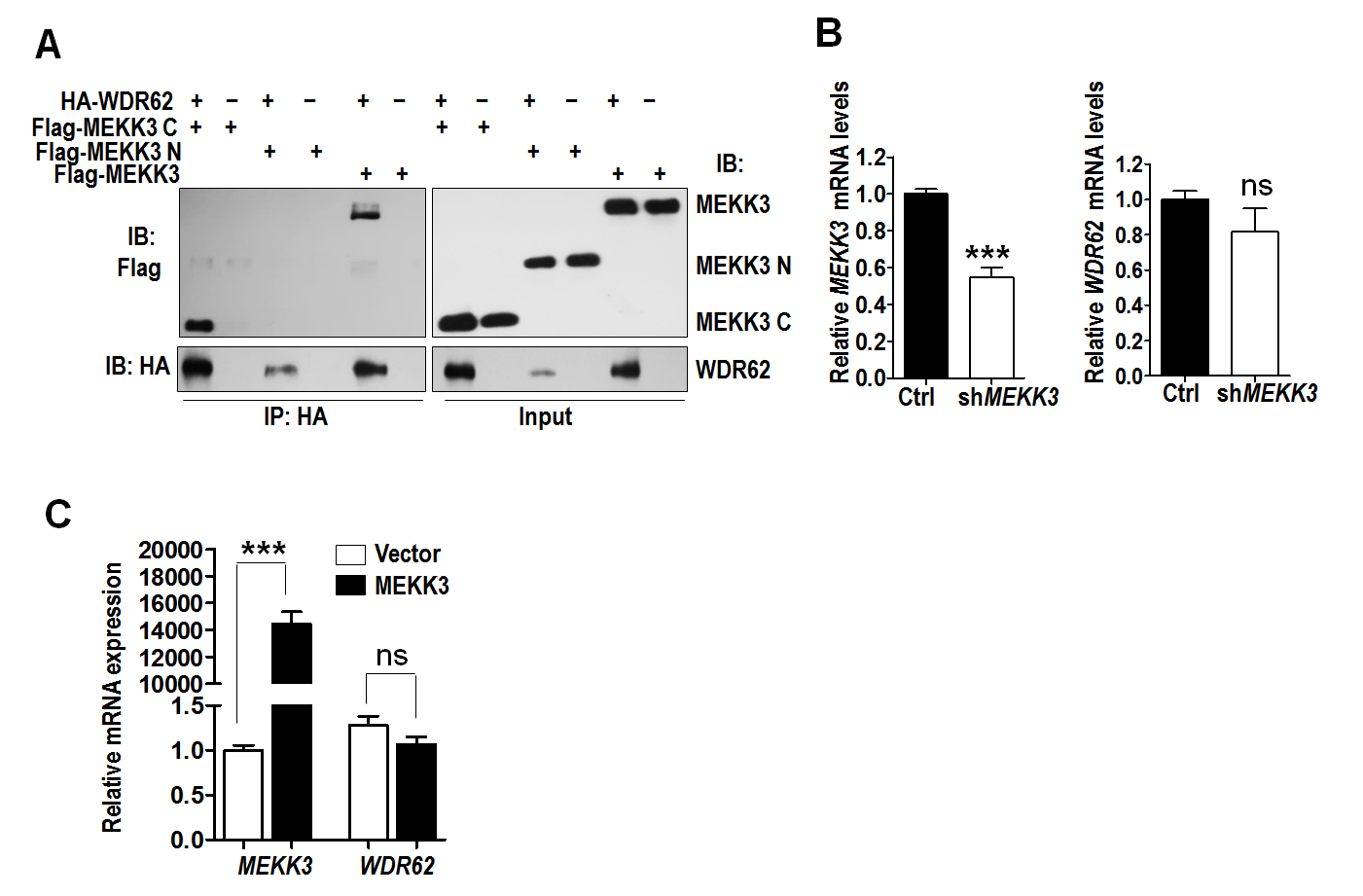

Supplement: S3 Fig — (A) Reciprocal immunoprecipitation of Fig 2E. (B) Relative WDR62 mRNA expression in MEKK3 KD cells. HEK293 cells were transfected with scramble control or human MEKK3 shRNA; 48 hours later, cells were collected for qPCR analysis. (C) Relative endogenous WDR62 mRNA expression in human MEKK3 overexpression cells. HEK293 cells were transfected with vector or HA-human MEKK3; 24 hours later, cells were collected for qPCR analysis. Underlying data can be found in S1 Data. HA, influenza hemagglutinin; IP, immunoprecipitation; MEKK3, mitogen-activated protein kinase kinase kinase 3; qPCR, quantitative PCR; shRNA, short hairpin RNA; WDR62, WD repeat domain 62. (TIF) [file pbio.2006613.s004.tif]

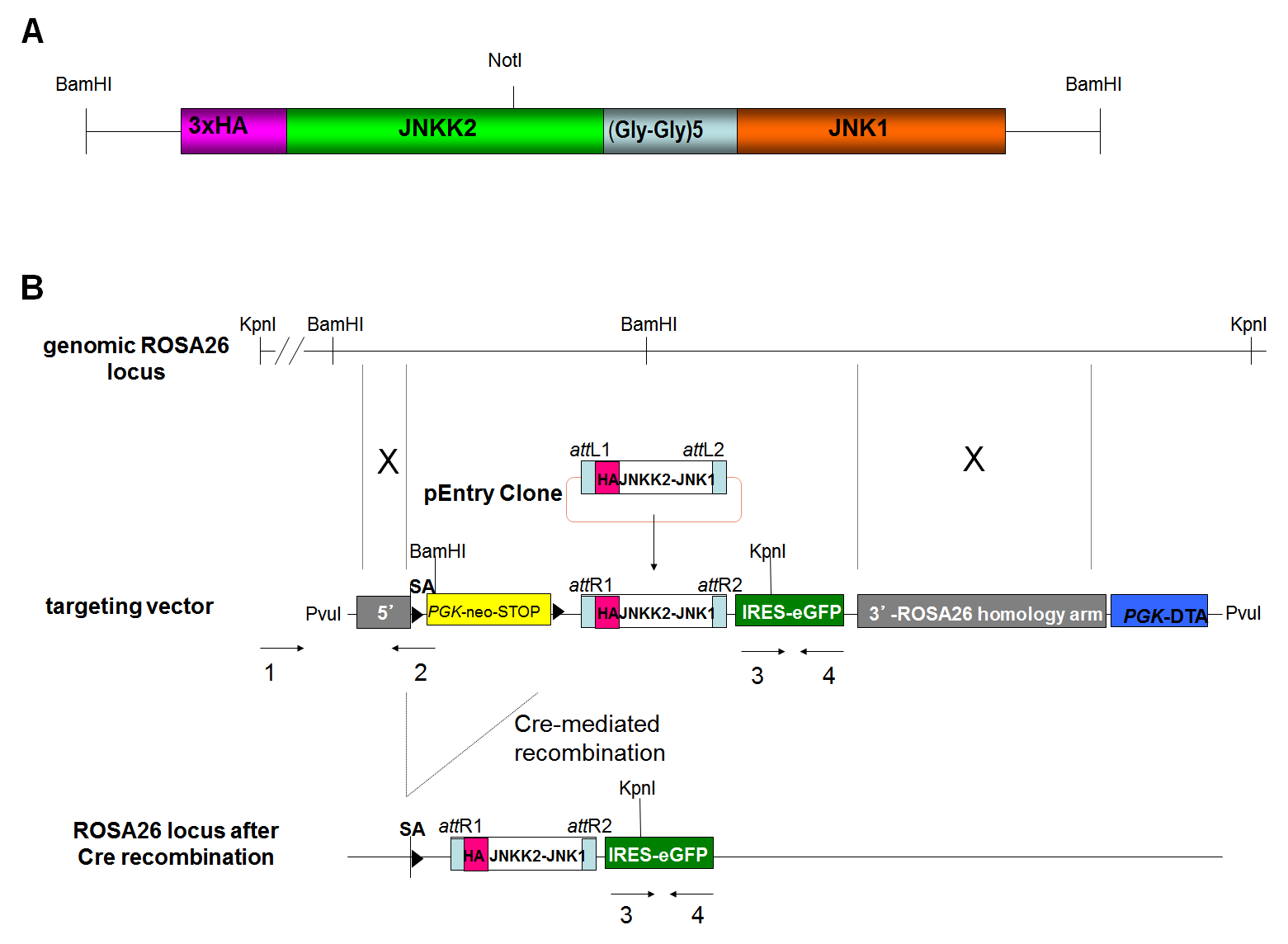

Supplement: S4 Fig — (A) Schematic of the JNKK2-JNK1 fusion constructs. The construct consists of a 3xHA tag, the human JNKK2 cDNA, (Gly-Gly) 5 repeats and the human JNK1 cDNA. The JNKK2-JNK1 fusion construct was inserted into the multiple cloning site of the pENTR1A vector (Life Technologies). (B) Targeting of the genomic ROSA26 locus with the JNKK2-JNK1 vector. In the pEntry clone, the JNKK2-JNK1 construct is flanked by lambda phage integrase recognition sites (attL) and thus can be efficiently inserted into the targeting vector carrying the corresponding heterotypic sites (attR). The targeting construct consists of a 5’-ROSA26 homology arm, a splice acceptor (SA) site, a PGK-neo-STOP cassette flanked by loxP-site (LSL), the JNKK2-JNK1 fusion construct, an IRES-eGFP reporter gene, a 3’-ROSA26 homology arm, and a PGK-DTA selection cassette. Screening PCR was performed using a forward primer indicated by arrow 1 and reverse primer indicated by arrow 2 in the 5’ region of the targeting construct. After Cre-mediated recombination, the LSL cassette is excised, and the JNKK2-JNK1 fusion construct is expressed in the genomic ROSA26 locus. For genotyping PCR, primers indicated by arrows 3, 4 located in the eGFP reporter gene were used. Arrowheads indicate loxP-sites. Underlying data can be found in S1 Data. eGFP, enhanced green fluorescent protein; HA, influenza hemagglutinin; IRES, internal ribosome entry site; JNK, c-Jun N-terminal kinase. (TIF) [file pbio.2006613.s005.tif]

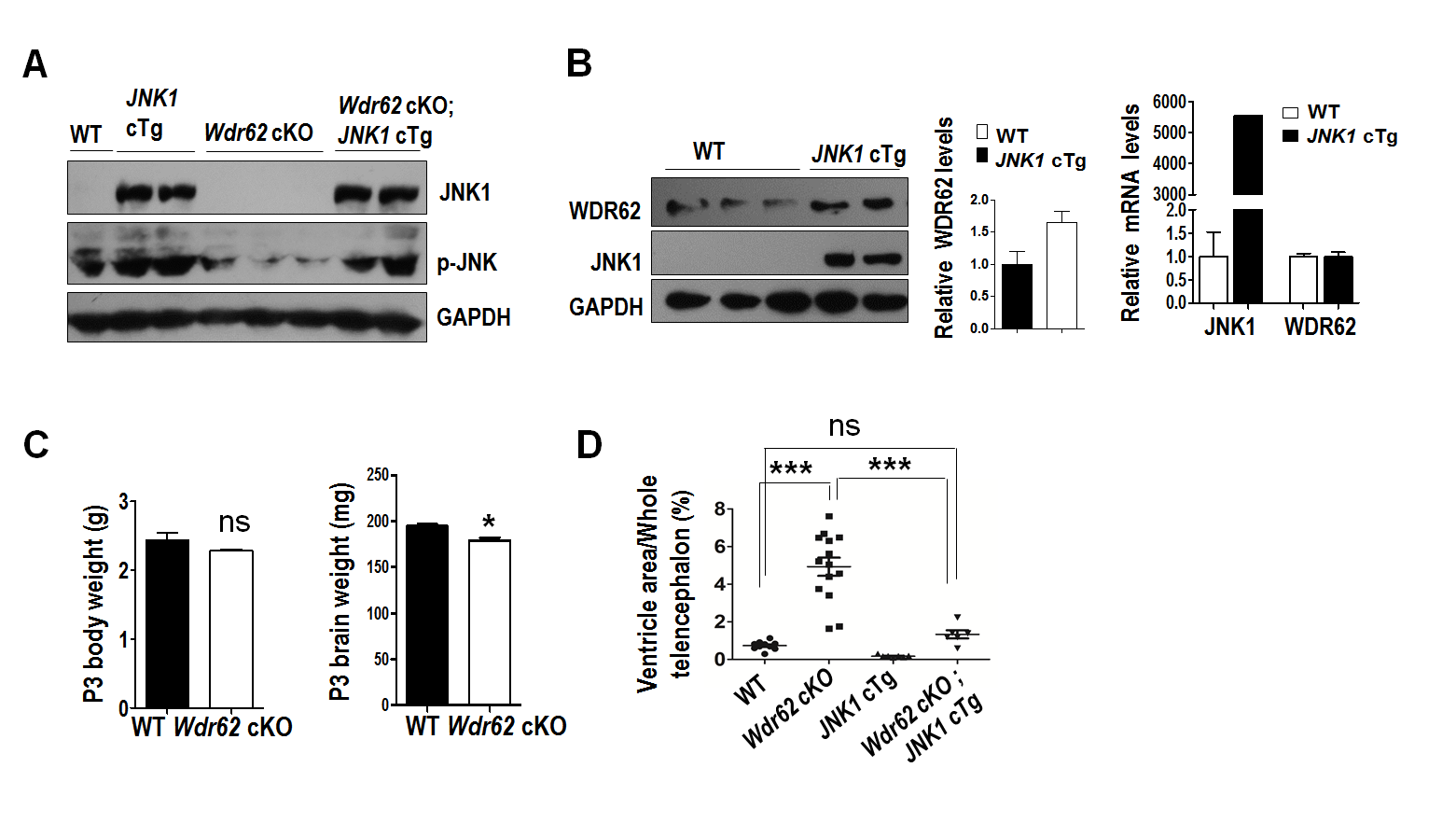

Supplement: S5 Fig — (A) Western blot analysis of JNK1 expression in the WT, JNK1 cTg, Wdr62 cKO, and Wdr62cKO;JNK1 cTg brains at E14.5. GAPDH was used as a loading control. (B) Western blot analysis of WDR62 expression in the E16.5 WT and JNK1 cTg mice brain. Right panels: quantification of WDR62 protein and mRNA expression. WT, n = 3; JNK1 cTg, n = 2. (C) Body and brain weight of P3 Wdr62flox/flox;Nestin-cre (Wdr62 cKO) and WT mice. Three Wdr62 cKO and WT littermates were analyzed. (D) Quantification of ventricle area as a percentage of whole telencephalon area. WT, n = 10; Wdr62 cKO, n = 14; JNK1 cTg, n = 7; Wdr62 cKO;JNK1 cTg, n = 6. n: brain numbers. All data are means ± SEM; ***P < 0.001, *P < 0.05, ns P > 0.05. Underlying data can be found in S1 Data. cKO, conditional knockout; E, embryonic day; JNK1, Jun N-terminal kinase 1; ns, not significant; WDR62, WD repeat domain 62; WT, wild-type. (TIF) [file pbio.2006613.s006.tif]

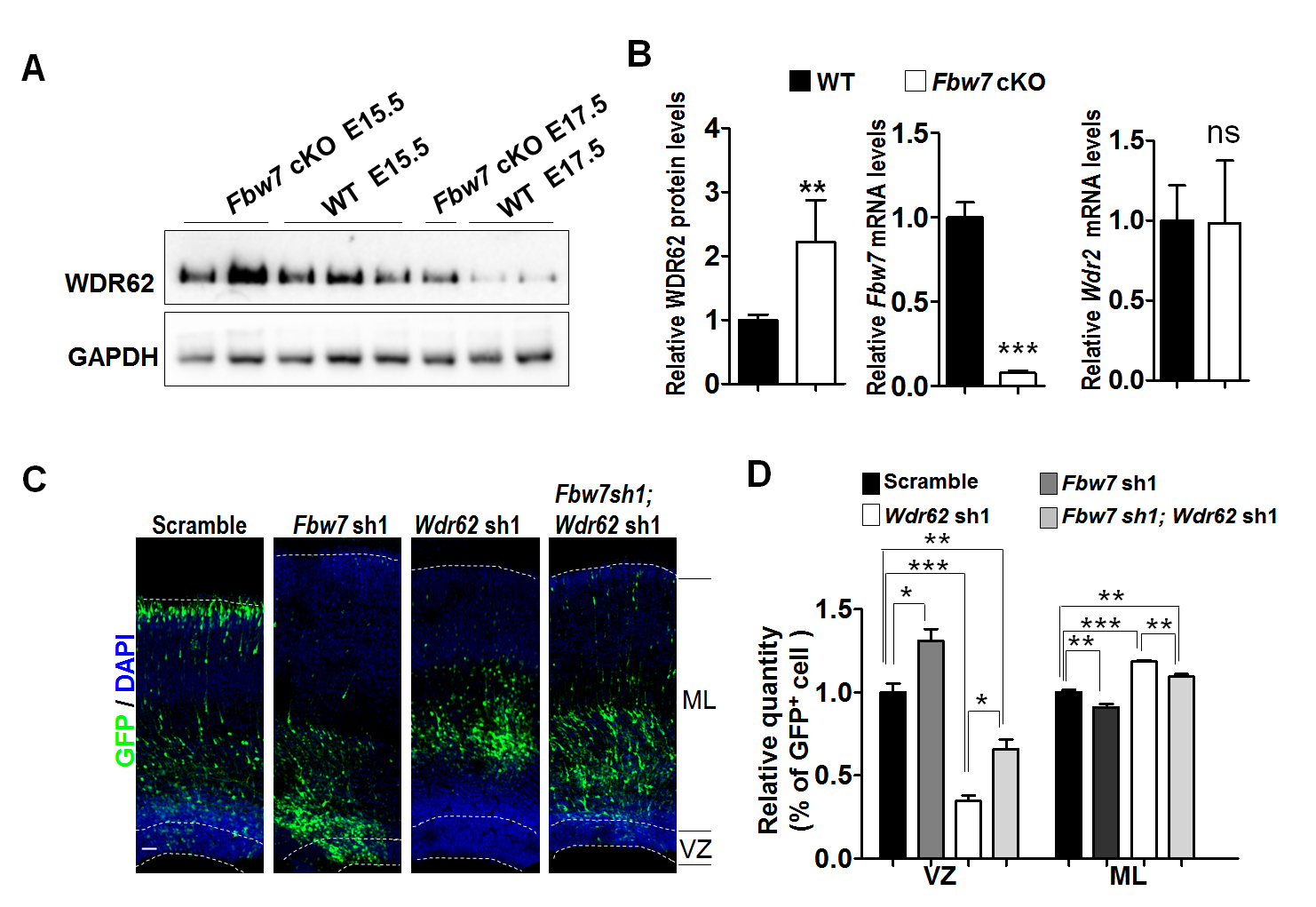

Supplement: S6 Fig — (A) E17.5 or E15.5 cortices from Fbw7 cKO and WT littermates were analyzed by western blot for endogenous WDR62 with GAPDH as control. (B) Left panel: quantification of WDR62 protein levels compared to WT control in panel A. Middle and right panel: relative Fbw7 and Wdr62 mRNA expression in 3 Fbw7 cKO and 5 WT mice. (C) Coronal sections of rat cortices electroporated in utero with bicistronic constructs encoding both EGFP and Wdr62 shRNA, Fbw7 shRNA or control shRNA (Ctrl) at E16.5 and inspected at E20.5. Scale bar 50 μm. In E20.5 cortex: “ML” indicates the mantle layer, including the cortical SVZ, IZ, and CP. (D) Relative quantity of cells in VZ and ML in panel C. Scramble, n = 6; Wdr62 shRNA1 (Wdr62sh1), n = 7; Fbw7 shRNA1 (Fbw7 sh1), fbw7 sh1; wdr62 sh1, n = 8. All data are means ± SEM; ***P < 0.001, **P < 0.01, *P < 0.05, ns P > 0.05. Underlying data can be found in S1 Data. CP, cortical plate; E, embryonic day; FBW7, F-box and WD repeat domain-containing protein 7; IZ, intermediate zone; ML, mantle layer; ns, not significant; SVZ, subventricular zone; VZ, ventricular zone; WDR62, WD repeat domain 62; WT, wild-type. (TIF) [file pbio.2006613.s007.tif]

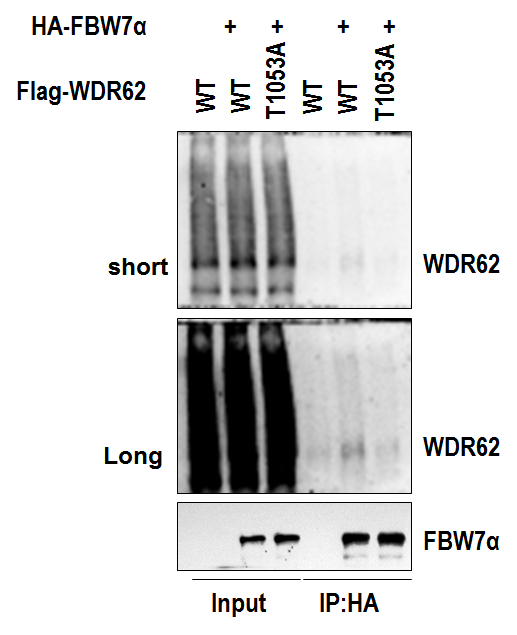

Supplement: S7 Fig — WDR62 T1053A showed weak interaction with FBW7 compared with WDR62 WT. HEK293 cells were transfected with Flag-WDR62 and Flag-WDR62-T1053A either alone or in combination with HA-FBW7α; 16 hours later, cells were treated with MG132 for 4 hours. Cell lysates were immunoprecipitated with HA antibody and probed with HA or WDR62 antibodies. FBW7, F-box and WD repeat domain-containing protein 7; HA, influenza hemagglutinin; WDR62, IP, immunoprecipitation; WD repeat domain 62. (TIF) [file pbio.2006613.s008.tif]
